# What Do Nectarivorous Bats Like? Nectar Composition in Bromeliaceae With Special Emphasis on Bat-Pollinated Species

**DOI:** 10.3389/fpls.2019.00205

**Published:** 2019-02-21

**Authors:** Thomas Göttlinger, Michael Schwerdtfeger, Kira Tiedge, Gertrud Lohaus

**Affiliations:** ^1^Molecular Plant Science and Plant Biochemistry, University of Wuppertal, Wuppertal, Germany; ^2^Albrecht-von-Haller-Institute for Plant Sciences, Georg-August-Universität Göttingen, Göttingen, Germany

**Keywords:** Bromeliaceae, floral nectar, flower ecology, nectar composition, bat-pollination, pollination syndrome

## Abstract

Floral nectar is the most important reward for pollinators and an integral component of the pollination syndrome. Nectar research has mainly focused on sugars or amino acids, whereas more comprehensive studies on the nectar composition of closely related plant species with different pollination types are rather limited. Nectar composition as well as concentrations of sugars, amino acids, inorganic ions, and organic acids were analyzed for 147 species of Bromeliaceae. This plant family shows a high diversity in terms of floral morphology, flowering time, and predominant pollination types (trochilophilous, trochilophilous/entomophilous, psychophilous, sphingophilous, chiropterophilous). Based on the analyses, we examined the relationship between nectar traits and pollination type in this family. Nectar of all analyzed species contained high amounts of sugars with different proportions of glucose, fructose, and sucrose. The total concentrations of amino acids, inorganic cations, and anions, or organic acids were much lower. The analyses revealed that the sugar composition, the concentrations of inorganic cations and anions as well as the concentration of malate in nectar of bat-pollinated species differed significantly from nectar of species with other pollination types. Flowers of bat-pollinated species contained a higher volume of nectar, which results in a total of about 25-fold higher amounts of sugar in bat-pollinated species than in insect-pollinated species. This difference was even higher for amino acids, inorganic anions and cations, and organic acids (between 50 and 100-fold). In general, bat-pollinated plant species invest large amounts of organic and inorganic compounds for their pollinators. Furthermore, statistical analyses reveal that the characteristics of nectar in Bromeliaceae are more strongly determined by the pollinator type rather than by taxonomic groups or phylogenetic relations. However, a considerable part of the variance cannot be explained by either of the variables, which means that additional factors must be responsible for the differences in the nectar composition.

## Introduction

The family of the Bromeliaceae is one of the species-richest non-woody plant families in the Neotropics. Additionally, it has experienced a remarkable adaptive radiation in the flora and, therefore, a wide variety of flower morphology has emerged (Benzing, 2000). The family is divided into eight subfamilies (Brocchinioideae, Lindmanioideae, Tillandsioideae, Hechtioideae, Navioideae, Bromelioideae, Puyoideae, Pitcairnioideae) which subsume approximately 58 genera and more than 3000 species (Givnish et al., 2011). The current taxonomie of Bromeliaceae is in strong flux due to newer morphological and genetic studies, so that new subfamilies or genera are created and species are frequently being assigned to other taxa (Zizka et al., 2013; Barfuss et al., 2016; Gomes-da-Silva and Souza-Chies, 2018). Bromeliads are adapted to various climates or other environmental conditions. Therefore, the plant family is also diverse in morphological, ecological, or physiological aspects; for example, about 60% of Bromeliaceae are epiphytic (Zotz, 2013) and several species use CAM photosynthesis to produce sugars (Crayn et al., 2015).

In bromeliads, as well as in other plant groups, floral nectar is the main reward for pollinators (Proctor et al., 1996), it is produced by septal nectaries (Sajo et al., 2004). Floral nectar is an aqueous solution rich in sugars as the major energy source for visitors. The main sugars in the nectar are the hexoses glucose and fructose and the disaccharide sucrose (Percival, 1961; Baker and Baker, 1983). The proportions of the three sugars can differ among the plant species so that there can be differentiated between hexose-rich and sucrose-rich nectars (Percival, 1961; Baker and Baker, 1983; Freeman et al., 1991; Perret et al., 2001; Nicolson and Fleming, 2003; Nicolson and Thornburg, 2007). Smaller amounts of other sugars, such as raffinose, are also present in some species (Witt et al., 2013; Lohaus and Schwerdtfeger, 2014).

Nectar also contains a wide range of amino acids, but, in general, their concentration is much lower than the sugar concentration (Baker and Baker, 1973). The biological significance of the amino acids is still being discussed. Amino acids in nectar are a potential nitrogen source for the floral visitors, therefore their concentration can affect the attractiveness of nectar (Baker and Baker, 1973). Phenylalanine, for example, has been shown to have a phagostimulatory effect on bees (Inouye and Waller, 1984). Furthermore, even low amino acid concentrations improve reproductive success of butterflies at suboptimal larval conditions (Mevi-Schutz and Erhardt, 2005). For avid insect-catchers, like most hummingbirds, however, amino acids in nectar are not essential, because they do not need an alternative nitrogen source (Baker, 1977).

Inorganic ions were also found in the nectar, with K^+^ being the most abundant cation and Cl^-^ the dominant anion (Hiebert and Calder, 1983; Nicolson and Worswick, 1990). A possible function of ions in nectar is to influence the electrolytic balance of the visitors (Hiebert and Calder, 1983). Organic acids as a component of floral nectars have only been, aside from initial studies showing their presence (Baker, 1977), considered in a few research studies (Noutsos et al., 2015; Tiedge and Lohaus, 2017).

In addition, other components like lipids, proteins, phenolic and other secondary compounds were identified in the nectar of some species (Baker, 1977; Seo et al., 2013; Zhang et al., 2016; Stevenson et al., 2017). The function of such compounds is to defend the nectar against robbers or microorganisms, as well as to attract pollinators (Adler, 2000; Sasu et al., 2010; Heil, 2011; Gosselin et al., 2013). Moreover, secondary metabolites such as nicotine and benzylacetone lead to a reduction of withdrawn nectar, but also to an increase of pollinators visits (Kessler and Baldwin, 2007).

The pollination syndrome is the adaptation of the floral morphology, color, scent, and even nectar composition to the preference of a specific group of pollinators (Fenster et al., 2004; Ashworth et al., 2015). In some cases, such as rare or absent visits of the primary pollinators, secondary pollinator groups may play an important role in plant reproduction (Rosas-Guerrero et al., 2014). Recently, the pollination syndrome concept has been criticized, as it is a fact that several flowers attract a broader spectrum of visitors (Ollerton et al., 2009; Schmid et al., 2011). However, flower visitation does not necessarily mean pollination (Waser et al., 1996; Souza et al., 2016). Different floral traits, like flower morphology, floral scent, duration of anthesis, time of pollen release, breeding system, and nectar compounds in relation to morphology and behavior of the floral visitors are considered to characterize the reproductive biology and the pollination type of plant species (Fumero-Cabán and Meléndez-Ackerman, 2007; Amorim et al., 2013). To determine the effectiveness of pollination, the frequency of visits and the quantity of the transferred pollen are analyzed, along with the quantity of fruits and seeds produced by the plant (Aguilar-Rodríguez et al., 2014). Such analyses were performed for some bat-pollinated bromeliad species, for example *Billbergia horrida.* Results show that although bats and hummingbirds visited the species, bats as nocturnal pollinators were much more related to the reproductive success of the bromeliad (Marques et al., 2015). Another species, *Tillandsia macropetala*, only produces nectar at night and bat-pollination resulted in the development of fruits (Aguilar-Rodríguez et al., 2014). Thus, the role of bats as effective pollinators has been elevated for different plant species.

At present, there are several research studies which indicate that bat- or sunbird-pollinated species produce nectar with high proportions of hexoses, whereas species which are pollinated by hummingbirds, butterflies, hawk moths, or long-tongued bees tend to produce sucrose-rich nectars (Baker and Baker, 1983; Baker et al., 1998; Galetto and Bernardello, 2003; Chalcoff et al., 2006; Schmidt-Lebuhn et al., 2007; Krömer et al., 2008; Aguilar-Rodríguez et al., 2016; Abrahamczyk et al., 2017). A number of studies also deal with the variability of amino acids in nectar relative to the pollination types (Baker and Baker, 1973; Petanidou et al., 2006; Bertazzini and Forlani, 2016), whereas studies which include different organic as well as inorganic nectar compounds are rather scarce (Tiedge and Lohaus, 2017).

More species within the Bromeliaceae are pollinated by vertebrates than insects, and most of them are pollinated by hummingbirds (Kessler and Krömer, 2000). Beside the trochilophilous (hummingbird-pollinated) species, bromeliads include chiropterophilous (bat-pollinated), entomophilous (insect-pollinated), and autogamous (self-pollinated) species (Kessler and Krömer, 2000; Krömer et al., 2006). Some studies indicate the existence of combined or generalist pollination systems (Aguilar-Rodríguez et al., 2016). Chiropterophilous species have been reported for different bromeliad genera, e.g., *Alcantarea, Billbergia, Encholorium, Guzmania, Pseudalcantarea, Pitcairnia, Puya, Tillandsia, Vriesea*, and *Werauhia* (Sazima et al., 1985; Cascante-Marín et al., 2005; Fleming et al., 2009; Aguilar-Rodríguez et al., 2014; Marques et al., 2015; Santos et al., 2017). Only 7% of 188 species in the Bolivian Andes and the adjacent lowland are bat-pollinated bromeliad species (Kessler and Krömer, 2000). The New World nectarivorous bats are members of the Glossophaginae (Fleming et al., 2009); they are relatively small (7.5–30 g body weight) with relatively long tongues compared to their overall size. All glossophagine bats can echolocate and, typically, hover when visiting flowers (Fleming et al., 2009). The greatest species richness of nectarivorous bats occurs in the wet tropical lowland forest.

To ensure nectar accessibility for the bats, nocturnal anthesis and an adapted morphology of the flowers are necessary. In order to attract these animals, the flower needs a contrasting color against the dark background at night (Tschapka and Dressler, 2002). In general, the color of the flowers is a brownish or greenish white (von Helversen and Winter, 2003). As known so far, bat-pollinated bromeliad species produce hexose-rich nectar (Krömer et al., 2008), and the nectar volume is in the upper range compared to species with other pollinators (Tschapka and Dressler, 2002). Amino acids in the floral nectar could also have an influence on the nectarivorous bats’ food selection (Rodríguez-Peña et al., 2013). However, bats generally supply their protein requirements with pollen and other plant parts and/or insects (Law, 1992; Herrera et al., 2001).

Nectar sugar compositions have often been related to the pollination syndrome of the plant species, including species of Bromeliaceae (Krömer et al., 2008; Schmid et al., 2011), whereas amino acids or other nectar compounds have not yet been investigated thoroughly. In the present study, nectars of 147 species from 18 genera of the Bromeliaceae were analyzed with regard to sugars, amino acids, inorganic ions (anions and cations), and organic acids. The analyzed species vary widely in the time of anthesis (day versus night), floral morphology, and pollination type. The investigation included 22 bat-pollinated species, which makes up 15% of the analyzed bromeliads. The main question of the study is whether the nectar composition is influenced by specific pollinator types, taxonomic groups, and/or phylogenetic relations. Furthermore, particular attention is paid to bat-pollinated species in pursuit of the question whether the nectar composition of bat-pollinated species differs fundamentally from the nectar of plants with other pollinators.

## Materials and Methods

### Plant Material and Collection of Nectar

The nectar samples were obtained from bromeliad plants grown in tropical glasshouses in the Botanical Garden and Botanical Museum Berlin (Germany), the Botanical Garden of the University of Bochum (Germany), the Botanical Garden of the University of Göttingen (Germany), the Botanical Garden of the University of Heidelberg (Germany), and the Botanical Garden of the University of Wien (Austria) between 2016 and 2018. All nectar samples were collected with the help of a micropipette on the first day of anthesis to minimize the effects of flower aging. A minimum of three nectar samples of different flowers from at least two different plants were collected from each of the 147 bromeliad species (Supplementary Table S1). The samples were stored at -80°C until analysis.

### Bromeliad Species, Flower Morphology and Pollinator Type

The 147 bromeliad species are members of 4 subfamilies and 18 genera: Bromelioideae (*Aechmea, Billbergia, Hohenbergia, Neoregelia, Nidularium, Quesnelia*), Puyoideae (*Puya*), Pitcairnioideae (*Deuterocohnia, Dyckia, Pitcairnia*), and Tillandsioides *(Alcantarea, Guzmania, Lemeltonia, Pseudalcantarea, Tillandsia, Vriesea, Wallisia*, and *Werauhia*). As it was possible to ascertain the pollination mode for only about half of the analyzed species from the literature, the other half of the analyzed species had to be classified via flower morphology and pollination syndrome (Supplementary Table S1). The analyzed bromeliads contain 107 trochilophilous, 8 trochilophilous/entomophilous, 8 psychophilous (butterfly-pollinated during the day), 2 sphingophilous (hawk moth-pollinated), and 22 chiropterophilous species.

The 22 chiropterophilous species are members of 8 genera (*Alcantarea, Guzmania, Pitcairnia, Pseudalcantarea, Puya, Tillandsia, Vriesea*, and *Werauhia*). As bat pollination was published for 15 of the 22 species, the other 7 species had to be classified via flower morphology and pollination syndrome (Supplementary Table S1). The flowers visited by bats generally have a matt-white color which can have a slight discoloration into green, brown, or yellow. The exception to this is the purple flower of *Tillandsia rauhii*, which may function more as a camouflage for other floral visitors and less as a visual attraction for bats (Fleming et al., 2009). The floral morphology can vary from large, wide open and cup-shaped to elliptical, small and tubular flowers. The corolla tube length differs between 15 and 80 mm, with the small flowers appearing in the genera *Guzmania* only, for example in *G. calothyrsus, G. farciminiformis*, and *G. killipiana*. Bromeliads with large cup-shaped or elliptical flowers open only one or two flowers at a time, whereas other species may open 3 or 4 in the same period.

### Influence of the Growth Site on the Nectar Composition

In order to test for a possible influence of the growth site on the nectar composition, the sugar concentrations in the nectar of *Pseudalcantarea grandis* from the botanical gardens Berlin and Heidelberg and that of *Aechmea racinae*, collected in the botanical gardens Berlin and Göttingen, were analyzed.

### Assay for Microbial Contamination

Microbial contamination was assayed according to the method of Tiedge and Lohaus (2017). Nectar samples were plated on malt extract as well as on lysogeny broth medium and they were incubated for one week at 28°C or 37°C.

### Analyses of Sugars in Nectar

The nectar sugars were analyzed via HPLC (Thermo Fisher Scientific Dionex ICS-5000+ HPIC System) according to Lohaus and Schwerdtfeger (2014). An anion exchange column (Dionex^TM^ CarboPac^TM^ PA10 4×250 mm; Dionex Corp, Sunnyvale, CA, United States) was eluted isocratically with 80 mM NaOH (Honeywell, United States). The sugars were detected by a pulse amperometric detector with gold electrode (ED cell, Dionex Corp, Sunnyvale, CA, United States). Sugar standards (Sigma-Aldrich, Darmstadt, Germany) were measured in parallel for external calibration. The measured chromatograms were evaluated with an integration program (Chromeleon 7.2, Dionex Corp, Sunnyvale, CA, United States). The sugar concentrations in the nectars were determined with the help of calibration curves for the different sugars. All sugar concentrations in the nectars are given in millimolar concentrations (mM). The millimolar sugar concentration can be multiplied by the molar mass of a given sugar to obtain mass concentrations (g L^-1^).

Nectar of the bromeliad species contained glucose, fructose, and sucrose. Based on the measurement results, the nectar samples of the species were defined and referred to as sucrose-rich or hexose-rich, with sucrose-rich meaning a proportion of sucrose higher than 50% and hexose-rich a proportion of glucose and fructose higher than 50%.

### Analyses of Free Amino Acids

The analysis of free amino acids was performed via HPLC according to Lohaus and Schwerdtfeger (2014). Amino acids with a primary amino group were processed by pre-column derivatization with o-phtaldialdehyde, amino acids with a secondary amino group (e.g., proline) were processed by pre-column derivatization with fluorenylmethyloxycarbonyl. Thereafter, the derivates were separated on the reversed-phase column (Merck, Darmstadt, Germany) with an acetonitrile gradient. The derivates were detected by fluorescence (derivatization with o-phtaldialdehyde: excitation 330 nm and emission 405 nm; derivatization with fluorenylmethyloxycarbonyl chloride: excitation 265 nm and emission 305 nm). Amino acid standards (Sigma-Aldrich, Darmstadt, Germany) were measured in parallel for external calibration. The evaluation of the chromatograms was performed with an integration program (Chromeleon 7.2, Dionex Corp, Sunnyvale, CA, United States). The concentration of the amino acids in the nectar was determined with the help of calibration curves for the different amino acids. All amino acid concentrations in nectars are given in millimolar concentrations (mM).

### Analyses of Inorganic Ions and Organic Acids

Anions and cations were analyzed separately via HPLC according to Lohaus et al. (2001). For the analysis of anions (inorganic anions and organic acids), an anion exchange column (IonPac^TM^ AS11 4 × 250 mm; Dionex Corp, Sunnyvale, CA, United States) and a sodium hydroxide gradient (4 to 77 mM in 30 min) was used. For the analysis of cations, a cation exchange column (CS 12A, 4 × 250 mm; Dionex Corp, Sunnyvale, CA, United States) with isocratic elution (20 mM H_2_SO_4_) was used. The ions were detected by their electronic conductivity (CP20 Conductivity Detector; Dionex Corp, Sunnyvale, CA, United States). Sugar standards were measured in parallel for external calibration. The measured chromatograms were evaluated with an integration program (Peaknet 5.1, Dionex Corp, Sunnyvale, CA, United States). The concentration of the inorganic ions in the nectar was determined with the help of calibration curves for the different inorganic ions. All concentrations of inorganic ions in nectars are given in millimolar concentrations (mM).

### Statistical Analysis of the Nectar Composition of All Analyzed Bromeliad Species

To analyze and compare the nectar compositions in different pollinator groups, an ANOVA followed by a *post hoc* test (Tukey) was carried out for all 147 bromeliad species (18 genera) (*p-*value < 0.05).

### Statistical Analysis of the Nectar Composition of 7 Genera With Bat-Pollinated Species

To analyze and compare the nectar compositions in bat-pollinated species and species with other pollination types the 7 genera (*Alcantarea, Guzmania, Pitcairnia, Puya, Tillandsia, Vriesea, Werauhia*), which contain bat-pollinated species as well as species with other pollinators, were selected. To analyze and compare the nectar compositions and the different components in different pollinator groups (bat-pollinator, residual-pollinator: hummingbirds, insects or butterflies), an ANOVA followed by a *post hoc* test (Tukey) was carried out (*p-*value < 0.05).

### Statistical Analysis of the Influence of Pollinator Type, Taxonomic Group, and Growth Site

A Principal Component Analysis (PCA) was conducted to examine the influence of the pollinator type and the taxonomic group (genera) on the nectar composition. 38 bromeliad species were included in this analysis: 19 bat pollinated species from 7 genera and the same number of hummingbird-pollinated species from the same 7 genera. The bat- and hummingbird-pollinated species per genus were from the same botanical garden. Prior to analysis, all data were normalized by *z*-transformation to set their means to zero and the variance to one. Five amino acids, which were not detectable in all the species, were removed from the data set. In the following PCA, two principal components were extracted from 25 initial variables. These variables were determined beforehand through measurements of sugars, amino acids, inorganic anions, inorganic cations, and organic acids. Varimax with Kaiser normalization was chosen as the rotation method in this procedure.

Furthermore, a Permutational Multivariate Analysis of Variance (PERMANOVA) was performed to identify the relative importance of the variables pollinator type (“Pollinator”) and taxonomic groups (“Genus”) on the nectar composition. In addition, the possible influence of the growth site on nectar composition (“Bot. garden”) has also been taken into account during the statistical analysis of the nectar composition. The analysis was performed using the “vegan” package with the *adonis* routine of the program “R,” so that distance matrices based on permutation tests could be used (Anderson, 2001; Oksanen et al., 2007). For the PERMANOVA, Euclidean distance measure and 999 permutations were applied. All statistical analyses were performed using R (version 3.5.1, www.r-project.org).

### Phylogenetic Analysis

A schematic and simplified phylogenetic tree of all analyzed Bromeliaceae species combining molecular and morphological findings was established. The schematic tree was created using Mesquite 3.51; it is based on 23 different phylogenetic investigations of different molecular findings (Faria et al., 2004; Barfuss et al., 2005, 2016; Givnish, 2007; Horres et al., 2007; Hornung-Leoni et al., 2008; Almeida et al., 2009; Rex et al., 2009; Schulte et al., 2009; Chew et al., 2010; Jabaily and Sytsma, 2010; Sass and Specht, 2010; Givnish et al., 2011, 2014; Gomes-da-Silva et al., 2012; Versieux et al., 2012; Escobedo-Sarti et al., 2013; Costa et al., 2015; Evans et al., 2015; Pinzón et al., 2016; Schütz et al., 2016; Gomes-da-Silva and Souza-Chies, 2018; Moura et al., 2018). The pollinator type and the nectar composition (sugar ratio, amino acid concentration, concentration of inorganic ions) were mapped on the species level in order to visualize the phylogenetic distribution of bat-pollination.

### Statistical Analysis of the Influence of Pollinator Type and Phylogenetic Relation

In order to evaluate the non-independence of the data due to shared ancestry the phylogenetic relation was verified by comparative analyses. First, two discrete (binary) traits were analyzed to obtain an evolutionary correlation. This implies investigating whether the absence or presence of one characteristic correlates with the absence or presence of another characteristic (Pagel, 1994). Therefore, to validate the correlation BayesTraits (version 3.0.1, www.evolution.rdg.ac.uk) was used since maximum likelihood and Bayesian methods are included. By this a discrete, dependent and independent model were performed to analyze the methods against each other to determine a likelihood ratio. For this the differences of the – log likelihood (Lh) of the two models was multiplied by 2 and this ratio was compared to a chi-square distribution. Further, a Phylogenetic Generalized Least Squares (PGLS) regression analysis was implemented to study, if phylogenetic relationship influences the similarity in species traits (phylogenetic signal). For the analyses, the branch lengths were generated by Grafen transformation (Grafen, 1989), in order to fit the assumptions of independent contrasts. In order to estimate the phylogenetic correlation between two characters, the factor lambda λ was estimated at the same time (kappa and delta = 1). Lambda λ can reach a value from 0 to 1. At lambda λ zero traits are independent of phylogeny resulting in a star phylogeny. Values between 0 and 1 indicate different levels of the phylogenetic signal. If the lambda λ reaches value 1, it can be assumed that the phylogeny predicts the distribution of the trait and a Brownian movement is present. PGLS analysis was calculated using the “caper” package (Orme et al., 2013) in R (version 3.5.2, www.r-project.org).

## Results

### Nectar Samples

A minimum volume of about 4–5 μL per nectar sample was required to analyze the different components, including sugars, amino acids, inorganic ions (anions and cations), and organic acids. Furthermore, a minimum of three independent samples per species was required for the analysis. Nectar samples of 147 species of a total number of 300 sampled species of Bromeliaceae fulfilled these criteria (Supplementary Table S1).

### Influence of the Growth Site on the Nectar Composition

**FIGURE 1:**
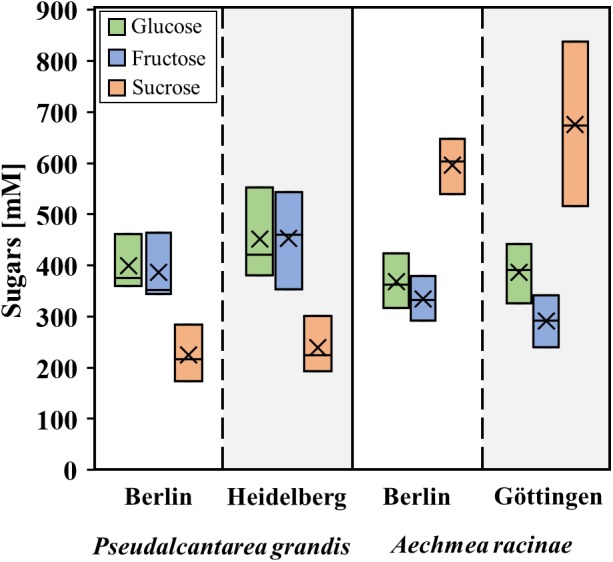
Boxplots of sugar composition in nectar from different botanical gardens. Comparison of one bat-pollinated (*Pseudalcantarea grandis*) and one hummingbird-pollinated (*Aechmea racinae*) species from two different botanical gardens. The sample number for each species was *n* = 3. The box plots show medians (horizontal line in box) and means (× in box).

In the present study, nectar samples of plants from different botanical gardens were used and it is conceivable that the growth site has an influence on the nectar composition. Therefore, the sugar concentrations of the same bromeliad species from different botanical gardens were analyzed (Figure 1). The sugar concentrations in the nectar of *Pseudalcantarea grandis* from the botanical gardens Berlin and Heidelberg showed no significant difference (*p* > 0.05, *df* = 5, *n* = 6). The same applies to the sugar concentrations in nectar of *Aechmea racinae*, collected in the botanical gardens Berlin and Göttingen.

### Microbial Contamination

In order to test for the possibility of the differences in sugar composition being a result of microbial activity, the samples were tested for the presence of yeast or bacteria. The test revealed no microbial contaminations in the nectar samples from bromeliad species with different pollinators.

### Sugar, Amino Acid, and Ion Concentrations in Nectar of All Species

**FIGURE 2:**
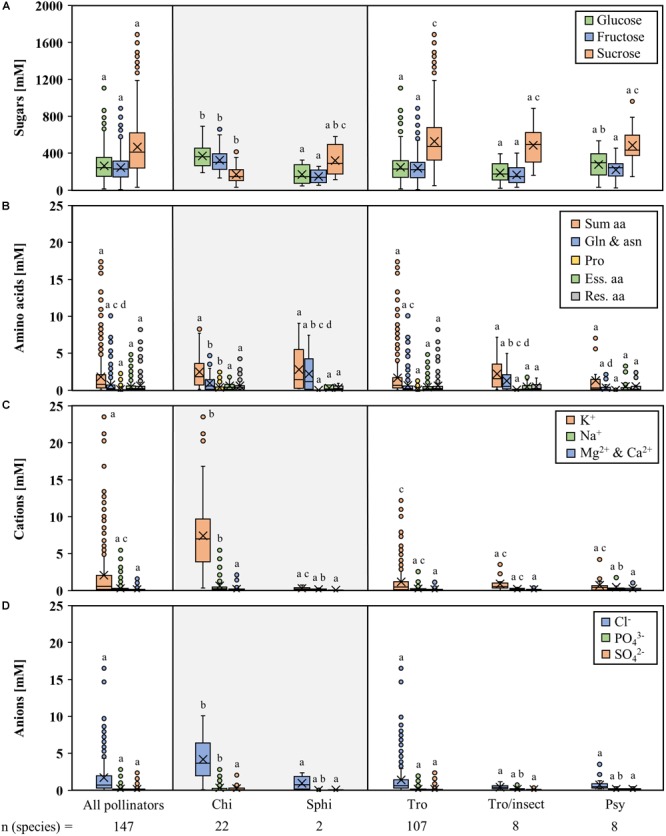
Concentrations of different compounds in nectar of all analyzed bromeliad species. The data are arranged by the nocturnal and diurnal pollinators of the species. The nocturnal bromeliads include 22 chiropterophilous and 2 sphingophilous species. The diurnal bromeliads include 107 trochilophilous, 8 trochilophilous/ entomophilous, and 8 psychophilous species. Boxplot diagrams illustrating the following components: sugars **(A)**, amino acids **(B)**, inorganic cations **(C)**, and inorganic anions **(D)**. The boxplots show medians (horizontal line in box) and means (× in box). Different letters represent significant differences in each sugars (glucose, fructose, sucrose), amino acids (sum amino acids, glutamine, and asparagine, proline, essential amino acids, residual amino acids), cations (potassium, sodium, magnesium, calcium), and anions (chloride, phosphate, sulfate) between the different pollination groups (Tukey’s HSD; *p* < 0.05).

The nectar of all 147 analyzed bromeliad species contained the three sugars glucose, fructose, and sucrose; no other sugars were detected in appreciable amounts (Figure 2A and Supplementary Table S2). Glucose and fructose were present in equal amounts in the nectar of every given species. The total sugar concentration (sum of glucose, fructose, and sucrose) was between 232 mM (*Tillandsia propagulifera*) and 2553 mM (*Nidularium scheremetiewii*).

The concentration of sugars differentiated by pollination type are shown in Figure 2A. The concentrations of hexoses and sucrose in the nectar of bat-pollinated species differed significantly from those of species with other pollination types (Figure 2A; *p* < 0.001, *df* = 876, *n* = 441). Nectar of bat-pollinated plant species contained more hexoses than sucrose, whereas the nectar of species with other pollination types was sucrose-rich throughout all samples, independent of the pollinator (hawk moths, hummingbirds, hummingbirds/insects, or butterflies). Therefore, the sucrose-to-hexoses ratio was significantly lower in bat-pollinated species than in the nectar of the other species (*p* < 0.001, *df* = 436, *n* = 441). In bat-pollinated species, the ratio was 0.5 ± 0.1, whereas in the other groups it ranged from 2.0 ± 0.0 (hawk moths) to 3.2 ± 1.5 (hummingbirds/insects).

In contrast to the high concentrations of sugar in the nectars of all samples, the concentrations of the 20 detected amino acids were at a rather low level (sum of amino acids 0.1 to 16.2 mM; Supplementary Table S3). The amino acid concentrations showed a much wider variability than was found for the sugar concentrations. The most abundant amino acids were glutamine and asparagine, followed by serine, alanine, and proline. Nevertheless, the proportions of these amino acids varied greatly depending on the plant species.

No significant differences between the total amino acid concentrations in the nectars of species with different pollinators could be shown (Figure 2B), whereas the concentration of amides (asparagine and glutamine) and proline differed between species with different pollination types.

The concentration of inorganic anions in nectar ranged from 0.1 to 16.9 mM, and chloride was the most abundant anion (Supplementary Table S4). The concentration of inorganic cations was in a similar range (0.1 to 22.5 mM) with potassium being the most abundant cation. Magnesium and calcium were detected in similar and low concentrations; therefore, the two cations were grouped together. The nectar of the bromeliad species also contained organic acids. In contrast to citrate, which was not detectable in most samples, malate could be detected more frequently and the concentration ranged from 0 up to 11 mM (Supplementary Table S4).

The inorganic cation and anion concentrations differentiated by pollination type are shown in Figure 2C,D. The nectar of bat-pollinated species showed significantly higher chloride and phosphate concentrations than the nectar of the other pollinators. In addition, significantly higher amounts of potassium and sodium were detectable.

### Nectar Composition in Species of Genera With Bat-Pollination

The results of the nectar composition of all 147 species imply that the composition of bat-pollinated species differs from all other pollination types. In a next step, only genera which include both chiropterophilous as well as other types of pollinators were considered. For this purpose, the seven genera *Alcantarea, Guzmania, Pitcairnia, Puya, Tillandsia, Vriesea*, and *Werauhia* were investigated more closely. The genus *Tillandsia* was recently divided in several genera (Barfuss et al., 2016). One of the resulting new genera, *Pseudalcantarea*, contains also bat-pollinated species; but in this case, no species with other types of pollinators are known.

**FIGURE 3:**
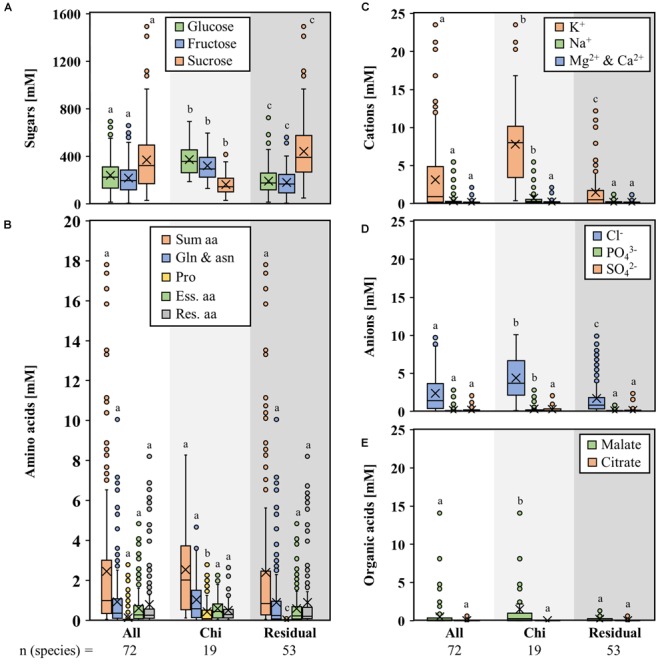
Concentrations of different compounds in nectar of seven genera with bat-pollinated species. Comparison of the nectar constituents of seven bromeliad genera (*Alcantarea, Guzmania, Pitcairnia, Puya, Tillandsia, Vriesea*, and *Werauhia*), which include bat-pollinated species as well as species with other pollination types. Boxplot diagrams illustrate the following components: sugars **(A)**, amino acids **(B)**, cations **(C)**, anions **(D)**, and organic acids **(E)**. All concentrations are given in mM. The boxplots show medians (horizontal line in box) and means (× in box). Different letters represent significant differences in each sugars (glucose, fructose, sucrose), amino acids (sum amino acids, glutamine, and asparagine, proline, essential amino acids, residual amino acids), cations (potassium, sodium, magnesium, calcium), anions (chloride, phosphate, sulfate), and organic acids (malate, citrate) between the different pollination groups (Tukey’s HSD; *p* < 0.05).

Figure 3A–E show the mean concentration of sugars, amino acids, inorganic cations, and anions as well as organic acids in nectar of bat-pollinated species and species with other pollination types from the seven genera mentioned above. The results for the single genera are shown in the Supplementary Figures S1–S5. The concentrations of glucose and fructose in nectar of bat-pollinated bromeliads were significantly higher than in species with other pollination types, whereas the sucrose concentration was significantly lower (Figure 3A). In the case of the amino acids, a significantly higher concentration in the nectar of bat-pollinated species was found for proline only (Figure 3B). The differences for the other amino acids were not on a significant level. A comparison of bat-pollinated species and the other species also showed that the concentrations of potassium, sodium, chloride, phosphate, and malate were significantly higher in the nectar of bat-pollinated species than those of species with other pollination types (Figure 3C–E).

### Amounts of Sugars, Amino Acids, and Ions in Nectar per Flower

**FIGURE 4:**
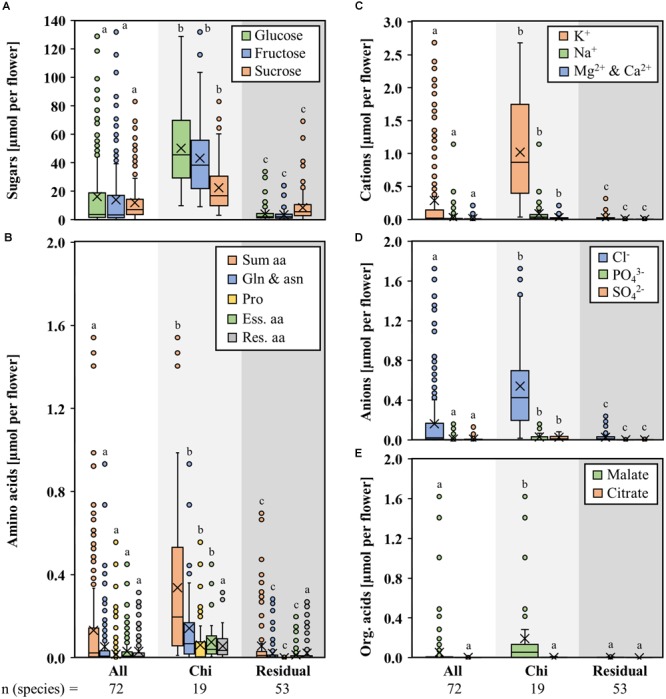
Amount of different nectar compounds per flower in seven genera with bat-pollinated species. Comparison of the nectar constituents of seven bromeliad genera (*Alcantarea, Guzmania, Pitcairnia, Puya, Tillandsia, Vriesea*, and *Werauhia*), which include bat-pollinated species as well as species with other pollination types. Boxplot diagrams illustrate the following components: sugar **(A)**, amino acids **(B)**, cations **(C)**, anions **(D)**, and organic acids **(E)**. All amounts are given in μmol per flower. The box plots show medians (horizontal line in box) and means (× in box). Different letters represent significant differences in each sugars (glucose, fructose, sucrose), amino acids (sum amino acids, glutamine, and asparagine, proline, essential amino acids, residual amino acids), cations (potassium, sodium, magnesium, calcium), anions (chloride, phosphate, sulfate), and organic acids (malate, citrate) between the different pollination groups (Tukey’s HSD; *p* < 0.05).

The flowers of the different bromeliad species contained different volumes of nectar. Very small nectar volumes were found in hawk moth-pollinated species (1–3 μL), small volumes in hummingbird/insect- and butterfly-pollinated species (3–10 μL), high nectar volumes in species pollinated by hummingbirds only (10–50 μL), and large nectar volumes in species pollinated by bats (up to 200 μL and more). Therefore, the total amounts of sugars, amino acids, inorganic ions, and organic acids per flower were also diverse in different bromeliad species (Figure 4A–E). The total amounts per flower were calculated using the nectar concentrations and the nectar volumes. When considering the total amount of compounds per flower, the differences between bat-pollinated bromeliads and those with other pollinators are more pronounced, with bat-pollinated species providing about 47 μmol each glucose or fructose, 22 μmol sucrose, 0.3 μmol amino acids, 1.1 μmol inorganic cations, 0.6 μmol inorganic anions, and 0.2 μmol malate. These amounts all are significantly higher than the corresponding amounts in flowers of species with other pollination types (sugars 7-fold, amino acids 6-fold, cations 40-fold, anions 22-fold, malate 71-fold).

### Nectar Composition in Relation to Pollination Type, Taxonomy, and Growth Site

In order to reduce the amount and complexity of the data, a PCA was performed to capture the complete diversity at once. Again, the focus was on the seven aforementioned genera, with all species visited by bats being included in the analyses. The same number of hummingbird-pollinated bromeliads from these genera was selected as well, as this pollination type is the most common in bromeliads; they are also from the same growth site.

**FIGURE 5:**
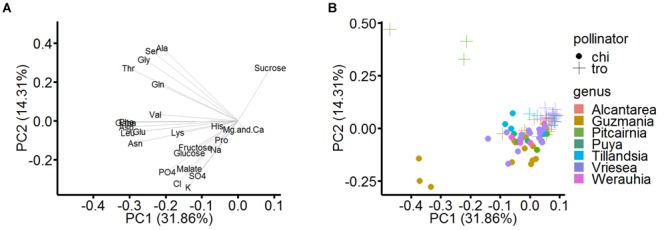
Loadings and scatterplot of PCA in rotated space (pollinator and taxonomic groups). In the PCA, the statistically analyzed samples were selected according to their pollinator and their taxonomic groups (genera). **(A)** Loading plot, which illustrate the original variables loaded as vectors in PCA space. The first principal component (PC 1) describes 31.9% and the second principal component (PC 2) describes 14.3% of the dataset variation. **(B)** Scatterplot of PCA, in which the data are grouped by pollinator (markings) and genus (colors).

Figure 5A shows the loading plot of the analyzed nectar, the loading on the extracted principal components is illustrated. Most of the amino acids load negatively on the first component; hexoses, proline, and inorganic ions load negatively on the first and second component. Sucrose is the only constituent which loads positively on both main components. The two principal components explained 46.2% of the total variance. The scatterplot of this PCA is shown in Figure 5B, the scores focus on the distribution by pollinator groups and genera at the same time. There is a visual separation of the different pollinator types: When a diagonal from top left to right separates the individual points of the pollinators, the bat-pollinated species (filled circles) are clustered below this line and the hummingbird-pollinated species (crosses) are nearly completely located above. The only exceptions are some trochilophilous species of the genus *Guzmania*, which appeared in the lower quadrants due to increased concentrations of inorganic ions.

To support the graphical evaluation, a PERMANOVA was performed with the same nectar data using pollination type (bat, hummingbird), taxonomic group (genus), and growth site (botanical garden) as categorical variables (Table 1). When considering all components (sugars, amino acids, inorganic cations and anions, organic acids), there is a high significance for the categories of pollinators (*p* < 0.01) with 41% of the data variation being explained by the pollinator, only 6% by the genus, and 2% by the growth site (botanical garden).

**Table 1:** Results of the PERMANOVA: Degrees of freedom (*df*), pseudo-F (F), R^2^, and *p–*values.

	Degrees of freedom (*df*)	Pseudo-F (F)	R^2^	*p–*value
*All components [mM]*				
Pollinator	1	85.89	0.41	0.001 ^∗∗∗^
Genus	6	2.13	0.06	0.024 ^∗^
Bot. garden	3	1.68	0.02	0.126
Pollinator × Genus	6	2.53	0.07	0.012 ^∗^
Pollinator × Bot. garden	3	1.92	0.03	0.077
Genus × Bot. garden	1	2.85	0.01	0.066
Pollinator × Genus × Bot. garden	1	14.37	0.07	0.001 ^∗∗∗^
Residuals	68		0.32	
Total	89		1.00	

*PERMANOVA describes the percentage of the influence of the pollinator, the genus, and the botanical garden on the nectar composition. ^∗^*p* < 0.05, ^∗∗∗^*p* < 0.001.*

When considering the components individually (Supplementary Figure S6 and Table S5), it turns out that the impact on the sugars (41%, *p* < 0.001) is much higher by the pollinators than it is by the genus (6%; *p* < 0.05). For amino acids, however, the opposite effect becomes apparent: 37% is explained by the genus and only 3% by the pollinator (*p* < 0.001). When only considering the inorganic ions and organic acids in the PERMANOVA, 34% of the data variance is influenced by the pollinators and 18% by the genus (*p* < 0.001).

### Phylogenetic Distribution of Bat-Pollinated Species

In order to visualize the phylogenetic relationships between the different taxa investigated in this study, a schematic and simplified tree was established (Supplementary Figure S7). About two thirds of the 147 bromeliad species could be phylogenetically classified on the basis of molecular findings. The analyzed bat-pollinated species were found in three subfamilies (Puyoideae, Pitcairnioideae, and Tillandsioideae). Bat-pollinated species are assigned a separate clade, like *Pseudalcantarea*, or a clade together with species with other pollinators (e.g., *Puya ferruginea* and *Puya densiflora*).

### Nectar Composition in Relation to Pollination Type and Phylogeny

The phylogeny could strongly impact the composition of nectar; therefore, comparative analyses were used to affirm the non-independence of the data due to shared ancestry of the plants. To analyze correlated evolution of discrete characters BayesTraits was used. Therefore, the pollination type was compared with the sucrose to hexose ratio. The -log likelihood of the dependent model was -40.31 and of the independent model was -68.16. Based on these values a likelihood ratio of 55.7 (*p* < 0.001) can be calculated. This ratio rejects the hypothesis that both traits evolve independently in favor that there is a strong correlation between the compared traits. Further evolutionary correlation studies by PGLS by determining the factor lambda λ were performed in order to verify the phylogenetic influence of the traits. For this purpose, respectively a component of the nectar was investigated with the pollinator types. The characters which are researched by PGLS are sucrose to hexoses ratio (λ = 0.094; *r^2^* = 0.29, *p* < 0.001), sugar per flower (λ = 0.141; *r^2^* = 0.72, *p* < 0.001), amino acid per flower (λ = 0.000; *r^2^* = 0.40, *p* < 0.001) and inorganic ions per flower (λ = 0.000; *r^2^* = 0.78, *p* < 0.001). In all cases the estimated lambda values are approximately 0 or exactly 0, whereby the observed variations were independent of phylogeny.

All in all, the PCA with all nectar constituents indicates an increased influence of the pollination type rather than the growth site and the taxonomic group on the data variation. The corresponding PERMANOVA confirmed these results. Sugars, inorganic ions, and organic acids are the components that show the highest variation depending on the pollination type. Furthermore, the phylogenetic comparative analyses by PGLS indicate roughly no influence of the phylogenetic signal on nectar characters.

## Discussion

### Stability of Nectar Compounds

The nectar composition of a given species is relatively constant and independent of the locations of the greenhouses and the growth site of the plants (Figure 1). This is in line with a former study that showed that greenhouse and field nectar samples are similar in sugar composition (Krömer et al., 2008; Supplementary Table S6). Whereas nectar, which is collected from species in the field, can contain pollinator carried microbes (Herrera et al., 2009; Fridman et al., 2012; Aizenberg-Gershtein et al., 2017), no contamination with yeast or bacteria was found in the greenhouse grown bromeliad species. In order to track any possible changes of the nectar sugar composition after sampling, nectar of at least one hummingbird- and one bat-pollinated species (*Aechmea fasciata, Alcantarea imperialis*) were measured immediately after sampling and 12 and 24 h later. The sugar concentrations did not change significantly during this period (data not shown). However, it could not be excluded that other nectar compounds are influenced by different environmental conditions or growth sites. Therefore, the potential influence of the growth site was included in the analyses of the data.

### Origin of Nectar Compounds

Nectar of all analyzed bromeliad species contained high concentrations of sugars (about 600 - 1000 mM), with the most predominant sugars being glucose, fructose, and sucrose. The sum of amino acids, inorganic anions or cations, and organic acids, in contrast, are detected at rather low millimolar concentrations (Figure 2).

Nectar with all its compounds is produced by and secreted from nectaries in a multi-stage process (Roy et al., 2017). The nectaries are supplied by the phloem and the discrepancy of solute composition and concentration between phloem sap and nectar has already been shown for different plant species (Lohaus and Schwerdtfeger, 2014; Tiedge and Lohaus, 2018). Although the total sugar concentrations of the phloem sap and the nectar are similar, hexoses only occur in the nectar and typically not in the phloem sap (Lohaus and Moellers, 2000; Lohaus and Schwerdtfeger, 2014). Therefore, the proportion of hexoses in nectar depends on the presence and activity of sucrose-cleaving enzymes, such as cell wall invertases (Ruhlmann et al., 2010; Tiedge and Lohaus, 2018). So far, it has not been possible to collect pure phloem sap from intact plants of Bromeliaceae like it was performed for other plant species. Therefore, the phloem compounds in the different bromeliad species have not yet been determined. Nevertheless, it is very likely that these species also translocate only sucrose in the phloem.

The nectar sugar concentration in day-flowering species was about 20% higher than in night-flowering species; however, this difference was not on a significant level (*p* = 0.087, *df* = 145, *n* = 147; Figure 2A and Supplementary Table S2). Similar results were shown for day- and night-flowering *Nicotiana* species (Tiedge and Lohaus, 2017). It could be a lower assimilation rate of carbon and a lower phloem translocation rate at night that cause these lower sugar concentrations in the nectar of night-flowering species (Riens et al., 1994). In addition, the higher humidity during the night could lead to slower evaporation (Witt et al., 2013).

The sugar-to-amino-acid-ratio as well as the sugar-to-cation or sugar-to-anion-ratio were lower in the phloem sap than in the nectar (Table 2; Lohaus and Moellers, 2000; Lohaus and Schwerdtfeger, 2014). This may be an indication for an active regulation mechanism in the nectaries and for a reduced secretion of amino acids or inorganic ions into the nectar (Lohaus and Schwerdtfeger, 2014). In general, the low concentrations of amino acids and inorganic ions in the nectar are more similar to the corresponding concentrations in other extracellular fluids, like the leaf apoplast or the xylem; in the symplast, the concentrations are usually higher (Lohaus et al., 2001).

**Table 2:** Ratios of different compounds in nectar of bromeliad species with different pollinators.

Pollination type	Sucrose/hexose	Sugars/amino acids	Sugars/cations	Sugars/anions
Chi	0.5 ± 0.1ˆa	919 ± 1028ˆa	143 ± 100ˆa	276 ± 225ˆa
Sphi	2.0 ± 0.0ˆa,b	1795 ± 2438ˆa,b	1583 ± 1110ˆa,b	1875 ± 2342ˆa,b
Tro	2.5 ± 1.4ˆb	2649 ± 3669ˆb	1952 ± 2323ˆb	1829 ± 2261ˆb
Tro/Ent	3.2 ± 1.5ˆb	1238 ± 1298ˆa,b	1034 ± 716ˆa,b	1488 ± 680ˆa,b
Psy	2.3 ± 1.3ˆb	3838 ± 4296ˆb	1446 ± 1218ˆa,b	1369 ± 906ˆa,b

*Data are calculated from the concentrations of sugars, amino acids, inorganic cations and anions in nectar of 147 bromeliad species (Supplementary Tables S2–S4). Pollinator: Chi, chiropterophilous; Sphi, sphingophilous; Tro, trochilophilous; Ent, entomophilous; Psy, psychophilous. Different letters represent significant differences between the different pollination type (Tukey’s HSD; *p* < 0.05).*

Furthermore, the amino acid composition can be affected by the growth conditions. It was shown, for example, that fertilization leads to higher proportions of glutamine, asparagine and proline in the nectar (Gardener and Gillman, 2001b). In addition, higher amino acid concentrations in the phloem sap of plants correlated with higher concentrations in the nectar (Lohaus and Schwerdtfeger, 2014). Many species of the Bromeliaceae are epiphytic, but there are also terrestrial species to be found; so it can be assumed that the different growing forms are influenced by the availability of nitrogen, which in turn influences the nitrogen content in the nectar. However, the concentration of amino acids in the nectar was not significantly different in the epiphytic and terrestrial plant groups (data not shown).

Variation of the water content in the nectar (concentrated versus diluted nectar) or of the nectar volume per flower (low versus high nectar volumes) can also be caused by biochemical variations in the nectaries (Nicolson and Thornburg, 2007). Furthermore, ambient humidity and the associated equilibrium has an influence on the nectar water content (Corbet, 2003). Hydrolysis of sucrose to glucose and fructose increases the osmolality of the nectar and subsequently the water flow from the nectaries to the nectar (Nicolson and Thornburg, 2007). With regard to the carbon equivalent per water equivalent in the nectar, it seems more effective to produce hexose-rich (monosaccharide with 6 carbon atoms) nectar instead of sucrose-rich (disaccharide with 12 carbon atoms) nectar. Bat-pollinated species produce high volumes of nectar (up to several hundred microliter nectar per flower) and the nectar is hexose-rich (Figure 2A, 3A). Similar results were shown for sunbird-pollinated *Nicotiana* species (Tiedge and Lohaus, 2017). However, results from *Brassica napus* are in contradiction to these findings, as its nectar was also dominated by hexoses but the nectar volume per flower was very low (Lohaus and Schwerdtfeger, 2014). Therefore, it is very probable that the nectar volume also depends on other ecological or physiological factors (Cresswell and Galen, 1991; Nicolson and Thornburg, 2007).

### Nectar Composition and Requirements of Pollinators

Nectar sugars represent the major energy source for pollinators (Percival, 1961) and the sugar composition in bromeliad species corresponded with their pollination type. Most of the hummingbird-pollinated species secreted sucrose-rich nectar, whereas the nectars of all analyzed bat-pollinated species were hexose-rich (Figure 2A, 3A and Supplementary Table S2). In an experimental study, Martínez del Rio (1990) demonstrated the preference for sucrose over hexoses for some hummingbird species. Generally, the nectar sugar preference of hummingbirds seems to be variable and it depends on the concentration of the offered sugar solution (Nicolson and Fleming, 2003). Similar to the nectar of bat-pollinated species, the nectar of sunbird-pollinated species is also often hexose-rich (Nicolson and Fleming, 2003; Tiedge and Lohaus, 2017). Moreover, it was shown that for passerine birds the sucrase activity was at a very low level and much lower than for example for hummingbirds (Martínez del Rio et al., 1992). For bat species, however, considerable sucrase activity has been detected (Schondube et al., 2001) and sucrose hydrolysis does not limit food intake in bats (Herrera and Mancina, 2008). Therefore, the reason for bat-pollinated species to produce hexose-rich nectar may not primarily be to serve the physiological needs of the bats.

Nectar is often a poor source of nitrogen (Baker and Baker, 1982) and for most pollinators it is not possible to rely solely on nectar for their nitrogen supply (Nicolson and Fleming, 2003). Pollen, fruits, or insects seem to serve as an additional nitrogen source for bats (Herrera et al., 2001; Mancina and Herrera, 2010). Hummingbirds also feed on insects, whereas nectarivorous butterflies lack an alternative nitrogen/protein source (Gilbert and Singer, 1975; Baker and Baker, 1982; López-Calleja et al., 2003). Despite that, all ten essential amino acids for most pollinators were present in the nectar of all our examined bromeliad species, albeit in varying proportions between 5 and 70% (Figure 2B and Supplementary Table S3). In most species, the predominant amino acid was glutamine, followed by asparagine, serine, alanine, and, in bat-pollinated species, also proline (Figure 2B and Supplementary Table S3). Similar amino acid compositions were shown for other plant species (Gardener and Gillman, 2001a; Tiedge and Lohaus, 2017). In contrast to the floral nectar of 73 Mediterranean plant species, where the proportion of phenylalanine was highly variable (Petanidou et al., 2006), the phenylalanine proportion was less variable and rather low in the nectar of all bromeliad species (about 2 ± 2%).

There are at least two possible reasons for the species-specific differences in the nectar composition of amino acids: (1) they are leaching from the nectaries and the nectar composition reflects the corresponding composition in the nectaries, or (2) the composition of amino acids in the nectar is adjusted to the preferences of different pollinators. Honey bees (*Apis mellifera*) and hummingbirds are attracted by proline in nectar (Waller, 1972; Quintana-Rodríguez et al., 2018). Proline-rich nectar seems to have a particular importance for insects such as Hymenoptera, because they use proline in the initial phase of flight (Carter et al., 2006; Nepi et al., 2012). Amino acids can also influence the food selection of bats (Rodríguez-Peña et al., 2013), but so far there is no knowledge about a preference for special amino acids.

The concentration of malate in the nectar was less than 0.3 mM in most non-bat-pollinated bromeliad species, whereas in the nectar of bat-pollinated species, the concentration was higher (1.6 ± 2.7 mM) but very variable (Figure 4E). An elevated concentration of malate was also found in *Nicotiana otophora*, a bat-pollinated species (Tiedge and Lohaus, 2017). It is likely that organic acids play a role in the attraction of pollinators by adding flavors to the nectar rather than constitute a carbon source for pollinators (Noutsos et al., 2015).

The nectar of bat-pollinated species is generally more dominated by inorganic ions than the nectar of species with other pollinators (inorganic anions are 2- to 6-fold higher and inorganic cations are 3-fold higher, Figure 2C,D). The processes leading to this difference in nectar composition are still poorly explored, but in general, the ion concentration in nectar influences the electrolyte balance of pollinators (Calder, 1979; Hiebert and Calder, 1983). The broad-tailed hummingbird (*Selasphorus platycercus*), for example, needs to replace 14% of its body electrolytes every day (Calder and Hiebert, 1983).

### Nectar Composition Is Influenced by Pollination Type Rather Than by Taxonomic Groups

The pollination modes vary between closely related species within the Bromeliaceae (Supplementary Figure S7; Kessler and Krömer, 2000; Krömer et al., 2008), and bat-pollinated species were found in several genera (Supplementary Table S1). The differing concentrations of sugars, amino acids, and inorganic ions in the nectars of species with different pollination types indicate that nectars of bat-pollinated species are different from the other, non-bat-pollinated species.

The analysis of the data shows that the pollination type (hummingbirds versus bats) has a much higher influence on the nectar composition than the plant genera or the growth site (Table 1). This applies when considering all measured nectar components (sugars, amino acids, inorganic ions, and organic acids), and also when only sugars or inorganic ions and organic acids were considered. However, if only amino acids are considered, the importance of pollinators over genera vanishes (Supplementary Table S5). Furthermore, there is an influence of the growth site on the inorganic ions and organic acids, but it is not very extensive at 7% (Supplementary Table S5). Similar analyses of *Nicotiana* species also revealed a strong influence of pollinator types on nectar composition, but here the pollinator type influenced the sugar and amino acid concentrations in the nectar more than the concentration of inorganic ions (Tiedge and Lohaus, 2017). However, the study included only one bat-pollinated species, which might explain the difference (Tiedge and Lohaus, 2017). Nevertheless, there is a considerable part of the variance that cannot be elucidated by either of the grouping options, which raises the question whether there are models or selective agents beyond this to predict the nectar composition (Parachnowitsch et al., 2018).

### Phylogenetic Distribution of Bat Pollination and Nectar Composition

Currently, there is no complete phylogeny of the family Bromeliaceae available due to neglecting or under-sampling of several species and genera. However, there are several models of the phylogeny based on the analyses of DNA and RNA sequences and sequence homologies. In 2013, a supertree for Bromeliaceae was established, but the authors noticed that at that time only about 20% of the bromeliad species have been included in phylogenetic analyses (Escobedo-Sarti et al., 2013). Since then, further studies have been published, but phylogeny still does not cover all species of this large family and particularly the larger genera (*Pitcairnia, Vriesea*, and *Tillandsia*) are still under-sampled (Palma-Silva et al., 2016). Therefore, a schematic phylogenetic tree of the analyzed bromeliad species was created based on published molecular studies (Supplementary Figure S7). The analyzed bat-pollinated species were found in the three subfamilies Puyoideae, Pitcairnioideae, and Tillandsioideae. In the subfamily Bromeliodeae, however, bat-pollination was not found for the analyzed species in this study (Supplementary Figure S7), although bat-pollination does exist in this subfamily as well (Marques et al., 2015).

Most of the bat-pollinated species evolved in addition to species with other pollination types (e.g., *Puya ferruginea* and *Puya densiflora*). Therefore, it is likely that in the family of Bromeliaceae, bat-pollinated species evolved several times alongside and from hummingbird-pollinated species (Benzing, 2000; Fleming et al., 2009; Aguilar-Rodríguez et al., 2014). Within the subfamily Tillandsioideae, however, there is a single clade including bat-pollinated species only, which may have arisen as a single evolutionary transition. The genus *Tillandsia* has recently been split into at least four new genera (Barfuss et al., 2016), among them the genus *Pseudalcanterea* (the former subgenus Pseudalcantarea), of which all known species are bat-pollinated. This might be an indication for an ongoing evolution from bird or moth pollination to bat pollination (Benzing, 2000; Aguilar-Rodríguez et al., 2014). Furthermore, Hoballah et al. (2007) demonstrated in their study that a major shift is caused by a single gene only, and thus the plants adapt to a new pollinator type. It can be assumed that the adaptation to bat-pollination is also reflected on the genetic level and with that on the taxonomic affiliation of a plant species and on the phylogenetic relations. However, further molecular phylogenetic analyses are necessary to understand the evolution of bat-pollination in this family.

The phylogenetic comparative analyses show that the trends in the nectar composition are not related to a phylogenetic signal. The maximum likelihood ratio by BayesTraits implicate a correlation between pollination type and sucrose to hexoses ratio. Further, the PGLS affirmed that there is roughly no phylogenetic signal in the analyzed data. These results corroborate the findings of Petanidou et al. (2006), or studies of Schmidt-Lebuhn et al. (2007) on Acanthaceae, but stand in contrast to, for example, findings of Nicolson and van Wyk (1998) for Proteaceae. This may be due to the fact that a putative phylogenetic constraint on nectar composition is more or less pronounced in different taxonomic groups (Krömer et al., 2008). In addition, it should be noted that the influence of the pollination type differs for the various nectar compounds. The sugar composition is clearly influenced by the pollination type, whereas, in the case of amino acids and inorganic ions, there is also an influence by the taxonomic group or by environmental conditions.

### Nectar Characteristics of Bat-Pollinated Species

Despite the fact that the total sugar concentrations in the nectar of species with different pollination types were roughly similar, the sugar composition was different and the nectar of bat-pollinated species showed lower sucrose-to-hexoses ratios (Table 2). It also contained more amino acids, inorganic ions, and organic acids than the nectar of species with other pollination types (Figure 3, 4) and the sugar-to-amino acids or sugar-to-ions ratios in the nectar of bat pollinated species were lower than in the nectar of the other bromeliad species (Table 2). In addition, bat-pollinated species produce higher volumes of nectar than non-bat-pollinated species. Therefore, the amount of sugars per flower was up to 25-fold higher in bat-pollinated species than in species with other pollination types (Figure 4A), the amount of amino acids per flower was up to 35-fold higher (Figure 4B), and the amount of inorganic ions per flower was up to several hundred-fold higher (Figure 4C,D). The difference was more pronounced between the nectar of bat- and that of insect-pollinated species than between the nectar of the two vertebrate pollinators, bats and hummingbirds. That means that bat-pollinated species invest large amounts of their carbohydrates, nitrogen compounds, and inorganic ions in this special pollination type. The benefit for the plant is that bats transfer large quantities of pollen and are long-distance pollen dispersers (Fleming et al., 2009).

A lot of the bat-pollinated bromeliad species belong to the C3 type of photosynthesis and not to the CAM type (Supplementary Table S1). In general, the CO_2_-assimilation rate per gram photosynthetic active tissue is higher in C3 plants than in CAM plants. One could assume that bromeliad species with CAM photosynthesis would not be able to afford losing high amounts of sugars through the nectar and, therefore, bat-pollination must be associated with C3 photosynthesis. In addition, C3 plants are common in more humid areas such as wet lowland regions, where chiropterophily is also most common (Kessler and Krömer, 2000).

Summarizing all data, it is obvious that significant differences exist between nectar traits of bat-pollinated species and species with other pollination types. However, all analyzed bat-pollinated bromeliad species in the Neotropics were pollinated by bat species of the Phyllostomidae (American leaf-nose bats), and the described nectar composition applies to this type of bat-pollination. Pollination by species of the Pteropodidae (Old World flying foxes) is also shown for plant species in Africa, Asia, and Australia (Fleming et al., 2009). Thus, more detailed studies of nectar traits are necessary to understand the adaptation of plants to pollination by New or Old World bats.

## Author Contributions

TG and GL conceived and designed the study, analyzed data, and wrote the manuscript. TG conducted experiments. TG and KT performed the statistical analysis. MS provided the plant material. All authors read and approved the manuscript.

## Conflict of Interest Statement

The authors declare that the research was conducted in the absence of any commercial or financial relationships that could be construed as a potential conflict of interest.
