# Supplementary material for: What Do Nectarivorous Bats Like? Nectar Composition in Bromeliaceae With Special Emphasis on Bat-Pollinated Species
Source: Front Plant Sci. 2019 Feb 21;10:205. doi: 10.3389/fpls.2019.00205 (PMC6393375; doi:10.3389/fpls.2019.00205)
Supplement: Supplementary file 1 [file Table_1.docx]

Supplementary Material

What do nectarivorous bats like? Nectar composition in Bromeliaceae with special emphasis on bat-pollinated species

**Author: Thomas Göttlinger, Michael Schwerdtfeger, Kira Tiedge, Gertrud Lohaus***

***Correspondence:** Gertrud Lohaus (lohaus@uni-wuppertal.de)

Supplementary Table S1: Overview of some main features of all examined Bromeliaceae species.

| **Species** | **Subfamily** | **Locality** | **Pollination type** | **Flower Color** | **Length of corolla tube [mm]** | **CAM or C3 (1)** |
| --- | --- | --- | --- | --- | --- | --- |
| *Aechmea abbreviata* L.B.Sm. | Brom. | Gö | Tro (2) | yellow | 15 | CAM |
| *Aechmea aquilega* (Salisb.) Griseb. | Brom. | Gö | Tro (3) | yellow | 20 | CAM |
| *Aechmea bruggeri* Leme | Brom. | Heid | Tro (4) | purple | 10 | CAM |
| *Aechmea cylindrata* Lindm. | Brom. | Boc | Tro (5) | yellow | 15 | CAM |
| *Aechmea distichantha* Lem. | Brom. | Ber | Tro (6) | purple | 10 | CAM |
| *Aechmea eurycorymbus* Harms | Brom. | Ber | Tro (2) | orange | 25 | CAM |
| *Aechmea fasciata* (Lindl.) Baker | Brom. | Wup | Tro (7) | purple | 35 | CAM |
| *Aechmea* *gamosepala* Wittm. | Brom. | Wup | Tro (5) | blue | 25 | CAM |
| *Aechmea gracilis* Lindm. | Brom. | Gö | Tro (2) | purple | 30 | CAM |
| *Aechmea leptantha* (Harms) Leme & J.A.Siqueira | Brom. | Heid | Tro (8) | yellow | 35 | CAM |
| *Aechmea marauensis* Leme | Brom. | Heid | Tro (2) | purple | 25 | CAM |
| *Aechmea miniata discolor* (Beer) Beer ex Baker | Brom. | Wup | Tro (9) | blue | 20 | CAM |
| *Aechmea nudicaulis* (L.) Griseb. | Brom. | Boc | Tro (5) | yellow | 15 | CAM |
| *Aechmea penduliflora* Andre | Brom. | Heid | Tro (2) | orange | 20 | CAM |
| *Aechmea pyramidalis* Benth. | Brom. | Boc | Tro (2) | white | 5 | CAM |
| *Aechmea racinae* L.B.Sm. | Brom. | Wup | Tro (9) | yellow | 15 | CAM |
| *Aechmea recurvata* (Klotzsch) L.B.Sm. | Brom. | Gö | Tro (5) | purple | 35 | CAM |
| *Aechmea weilbachii* Didr. | Brom. | Gö | Tro (9) | purple | 20 | CAM |
| *Alcantarea geniculata* (Vell.) Harms | Till. | Heid | Tro (2) | yellow | 40 | C3 |
| ***Alcantarea imperialis* (Carriere) Harms** | **Till.** | Heid | **Chi (10)** | **matt-white** | **80** | **C3-CAM** |
| *Billbergia amoena* (G.Lodd.) Lindl. | Brom. | Gö | Tro (5) | green | 45 | CAM |
| *Billbergia brasiliensis* L.B.Sm. | Brom. | Gö | Tro (2) | blue | 25 | CAM |
| *Billbergia buchholtzii* Mez | Brom. | Heid | Tro (2) | purple | 35 | CAM |
| *Billbergia distachia* (Vell.) Mez | Brom. | Gö | Tro 3 | green | 50 | CAM |
| *Billbergia euphemiae* E.Morren | Brom. | Heid | Tro (11) | blue | 40 | C3 |
| *Billbergia fosteriana* L.B.Sm. | Brom. | Gö | Tro (2) | blue | 60 | CAM |
| *Billbergia morelii Brongn.* | Brom. | Wup | Tro (8) | blue | 50 | CAM |
| *Billbergia nutans* H.Wendl. ex Regel | Brom. | Wup | Tro (2) | green | 40 | CAM |
| *Billbergia pyramidalis* (Sims) Lindl. | Brom. | Gö | Tro (8) | red | 40 | CAM |
| *Billbergia reichardtii* Wawra | Brom. | Heid | Tro (2) | purple | 35 | CAM |
| *Billbergia viridiflora* H.Wendl. | Brom. | Ber | Tro (3) | green | 50 | CAM |
| *Billbergia vittata* Brongn. ex C.Morel | Brom. | Gö | Tro (12) | blue | 50 | CAM |
| *Deuterocohnia brevispicata* Rauh & L.Hrom. | Pitc. | Ber | Tro /Ent (13) | pink | 25 | CAM |
| *Deuterocohnia longipetala* (Baker) Mez | Pitc. | Ber | Tro /Ent (2) | pink | 40 | CAM |
| *Deuterocohnia meziana subsp. carmineoviridiflora (Rauh) N.Schutz* | Pitc. | Heid | Tro /Ent (2) | green | 50 | CAM |
| *Deuterocohnia recurvipetala* E.Gross | Pitc. | Heid | Tro /Ent (2) | yellow | 10 | CAM |
| *Dyckia choristaminea* Mez | Pitc. | Heid | Tro /Ent (14) | yellow | 10 | CAM |
| *Dyckia goehringii* E.Gross & Rauh | Pitc. | Heid | Tro /Ent (14) | orange | 10 | CAM |
| *Dyckia leptostachya* Baker | Pitc. | Heid | Tro /Ent (14) | yellow | 10 | CAM |
| *Dyckia vestita* Hassl. | Pitc. | Heid | Tro /Ent (14) | yellow | 10 | CAM |
| *Guzmania acorifolia* (Griseb.) Mez | Till. | Ber | Tro (2) | white | 15 | C3 |
| ***Guzmania calothyrsus* Mez** | **Till.** | **Wup** | **Chi (15)** | **matt-white** | **15** | **C3** |
| *Guzmania conifera* (André) André ex Mez | **Till.** | Gö | Tro (2) | yellow | 30 | C3 |
| ***Guzmania cylindrica* L.B.Sm.** | **Till.** | **Gö** | **Chi (2)** | **matt-white** | **50** | **C3** |
| ***Guzmania farciminiformis* H.Luther** | **Till.** | **Gö** | **Chi (2)** | **matt-white** | **15** | **C3** |
| ***Guzmania killipiana* L.B.Sm.** | **Till.** | **Wup** | **Chi (9)** | **matt-white** | **30** | **C3** |
| *Guzmania lingulata* (L.) Mez | Till. | Heid | Tro (16) | yellow | 50 | C3 |
| *Guzmania melinonis* Regel | Till. | Gö | Tro (15) | yellow | 30 | C3 |
| *Guzmania monostachia* (L.) Rusby ex Mez | Till. | Wup | Tro (3) | white | 25 | C3-CAM |
| *Guzmania osyana* (E.Morren) Mez | Till. | Gö | Tro (2) | yellow | 35 | C3 |
| *Guzmania rhonhofiana* Harms | Till. | Wien | Tro (2) | white | 25 | C3 |
| *Guzmania roseiflora* Rauh | Till. | Boc | Tro (2) | yellow | 15 | C3 |
| *Guzmania sanguinea* (Andre) Andre ex Mez | Till. | Gö | Tro (2) | yellow | 75 | C3 |
| *Guzmania variegata* L.B.Sm. | Till. | Ber | Tro (2) | red | 30 | C3 |
| *Guzmania wittmackii* (Andre) Andre ex Mez | Till. | Gö | Tro (2) | white | 90 | C3 |
| *Guzmania zahnii* (Hook.f.) Mez | Till. | Wup | Tro (9) | yellow | 30 | C3-CAM |
| *Hohenbergia correia-araujoi* E.Pereira & Moutinho | Brom. | Ber | Tro (2) | yellow | 10 | CAM |
| *Hohenbergia leopoldo-horstii* E.Gross. Rauh & Leme | Brom. | Gö | Tro (2) | purple | 10 | CAM |
| *Hohenbergia rosea* L.B.Sm. & Read | Brom. | Heid | Tro (2) | purple | 10 | CAM |
| *Hohenbergia stellata* Schult. & Schult.f. | Brom. | Ber | Tro (2) | purple | 10 | CAM |
| *Hohenbergia utriculosa* Ule | Brom. | Ber | Tro (2) | purple | 10 | CAM |
| *Lemeltonia narthecioides* (C.Presl) Barfuss & W.Till | Till. | Gö | Sphi (2) | white | 10 | C3-CAM |
| *Lemeltonia scaligera* (Mez & Sodiro) Barfuss & W.Till | Till. | Gö | Sphi (2) | white | 20 | C3 |
| *Neoregelia ampullacea* (E.Morren) L.B.Sm. | Brom. | Boc | Tro (11) | purple | 15 | CAM |
| *Neoregelia carolinae* (Beer) L.B.Sm. | Brom. | Wup | Psy (2) | purple | 35 | CAM |
| *Neoregelia compacta* (Mez) L.B.Sm. | Brom. | Gö | Tro (11) | pink | 15 | CAM |
| *Neoregelia farinosa* (Ule) L.B.Sm. | Brom. | Gö | Psy (2) | purple | 50 | CAM |
| *Neoregelia fosteriana* L.B.Sm. | Brom. | Gö | Tro (2) | purple | 20 | CAM |
| *Neoregelia johannis* (Carrière) L.B.Sm. | Brom. | Ber | Tro (8) | white | 25 | CAM |
| *Neoregelia kautskyi* E.Pereira | Brom. | Gö | Tro (2) | white | 20 | CAM |
| *Neoregelia laevis* (Mez) L.B.Sm. | Brom. | Heid | Tro (2) | white | 20 | CAM |
| *Neoregelia martinellii* W.Weber | Brom. | Ber | Tro (2) | white | 20 | CAM |
| *Neoregelia olens* (Hook.f.) L.B.Sm. | Brom. | Gö | Psy (2) | purple | 40 | CAM |
| *Neoregelia pineliana* (Lem.) L.B.Sm. | Brom. | Ber | Psy (2) | purple | 40 | CAM |
| *Neoregelia seideliana* L.B.Sm. & Reitz | Brom. | Gö | Psy (2) | purple | 40 | CAM |
| *Neoregelia wilsoniana* M.B.Foster | Brom. | Gö | Tro (2) | white | 30 | CAM |
| *Nidularium amazonicum* (Baker) Linden & É.Morren ex Lindm. | Brom. | Wup | Tro (2) | white | 20 | C3-CAM |
| *Nidularium innocentii* Lem. | Brom. | Gö | Tro (5) | white | 50 | C3 |
| *Nidularium procerum* Lindm. | Brom. | Wup | Tro (7) | blue | 20 | CAM |
| *Nidularium purpureum* Beer | Brom. | Wup | Tro (2) | pink | 20 | CAM |
| *Nidularium rutilans* É.Morren | Brom. | Gö | Tro (5) | pink | 10 | CAM |
| *Nidularium scheremetiewii* Regel | Brom. | Gö | Tro (11) | blue | 55 | CAM |
| *Nidularium utriculosum* Ule | Brom. | Wup | Tro (2) | blue | 30 | CAM |
| *Pitcairnia bromeliifolia* L\'Heritier | Pitc. | Gö | Tro (2) | yellow | 40 | C3 |
| *Pitcairnia chiapensis* Miranda | Pitc. | Gö | Tro (2) | yellow | 40 | C3-CAM |
| *Pitcairnia chiriquensis* L.B.Sm. | Pitc. | Ber | Tro (2) | orange | 45 | C3 |
| *Pitcairnia grafii* Rauh | Pitc. | Ber | Tro (2) | orange | 50 | C3 |
| ***Pitcairnia recurvata* (Scheidw.) K.Koch** | **Pitc.** | **Ber** | **Chi (2)** | **matt-white** | **80** | **C3** |
| *Pitcairnia rubronigriflora* Rauh | Pitc. | Ber | Tro (2) | red | 50 | C3 |
| *Pitcairnia sprucei* Baker | Pitc. | Ber | Tro (5) | red | 60 | C3 |
| *Pitcairnia suaveolens* Lindl. | Pitc. | Ber | Tro (2) | yellow | 50 | C3 |
| *Pitcairnia utcubambensis* Rauh | Pitc. | Wien | Tro (2) | red | 60 | C3 |
| *Pitcairnia xanthocalyx* Mart. | Pitc. | Ber | Tro (2) | yellow | 45 | C3 |
| ***Pseudalcantarea grandis* (Schltdl.) Pinzón & Barfuss** | **Pitc.** | **Ber** | **Chi (17)** | **matt-white** | **90** | **C3** |
| ***Pseudalcantarea macropetala* (Wawra) Pinzon & Barfuss** | **Pitc.** | **Gö** | **Chi (18)** | **matt-white** | **30** | **C3-CAM** |
| ***Pseudalcantarea viridiflora* (Beer) Pinzón & Barfuss** | **Pitc.** | **Gö** | **Chi (7)** | **matt-white** | **50** | **C3** |
| *Puya coerulea var. violacea* (Brongn.) L.B.Sm. & Looser | Pitc. | Heid | Tro (11) | blue | 45 | C3-CAM |
| *Puya densiflora* Harms | Pitc. | Heid | Tro (2) | purple | 80 | CAM |
| ***Puya ferruginea* (Ruiz & Pav.) L.B.Sm.** | **Pitc.** | **Heid** | **Chi (15)** | **matt-white** | **80** | **C3-CAM** |
| *Puya spathacea* (Griseb.) Mez | Pitc. | Heid | Tro (11) | blue | 30 | C3 |
| *Quesnelia edmundoi* L.B.Sm. | Brom. | Gö | Tro (2) | white | 20 | CAM |
| *Quesnelia lateralis* Wawra | Brom. | Gö | Tro (2) | blue | 35 | CAM |
| *Quesnelia quesneliana* (Brongn.) L.B.Sm. | Brom. | Gö | Tro (19) | purple | 30 | CAM |
| *Tillandsia achyrostachys* E.Morren | Till. | Gö | Tro (20) | green | 55 | CAM |
| *Tillandsia aeranthos* (Loisel.) Desf. | Till. | Gö | Tro (21) | purple | 20 | CAM |
| *Tillandsia caput-medusae* E.Morren | Till. | Gö | Tro (2) | purple | 40 | CAM |
| *Tillandsia circinnatoides* Matuda | Till. | Gö | Tro (2) | purple | 40 | CAM |
| *Tillandsia clavigera* Mez | Till. | Heid | Tro (2) | purple | 20 | C3 |
| *Tillandsia concolor* L.B.Sm. | Till. | Gö | Tro (2) | pink | 60 | CAM |
| *Tillandsia flabellata* Baker | Till. | Gö | Tro (9) | purple | 50 | CAM |
| *Tillandsia foliosa* M.Martens & Galeotti | Till. | Gö | Tro (2) | purple | 40 | CAM |
| *Tillandsia funckiana* Baker | Till. | Wien | Tro (2) | red | 45 | CAM |
| *Tillandsia gerdae* Ehlers | Till. | Gö | Tro (2) | purple | 45 | CAM |
| ***Tillandsia heterophylla* E.Morren** | **Till.** | **Gö** | **Chi (22)** | **matt-white** | **70** | **C3** |
| *Tillandsia ionantha* Planch. | Till. | Gö | Tro (2) | purple | 50 | CAM |
| *Tillandsia ixioides* Griseb. | Till. | Gö | Tro (2) | yellow | 25 | CAM |
| *Tillandsia makoyana* Baker | Till. | Heid | Tro (2) | purple | 35 | CAM |
| *Tillandsia malzinei* (E.Morren) Baker | Till. | Gö | Tro (2) | white | 50 | C3 |
| *Tillandsia polystachia* (L.) L. | Till. | Gö | Tro (2) | purple | 40 | CAM |
| *Tillandsia ponderosa* L.B.Sm. | Till. | Heid | Tro (9) | purple | 65 | C3-CAM |
| *Tillandsia propagulifera* Rauh | Till. | Heid | Tro (2) | purple | 30 | CAM |
| ***Tillandsia rauhii* L.B.Sm.** | **Till.** | **Ber** | **Chi (2)** | **purple** | **90** | **C3-CAM** |
| *Tillandsia roland-gosselinii* Mez | Till. | Boc | Tro (2) | purple | 30 | CAM |
| *Tillandsia tricolor* Schltdl. & Cham. | Till. | Ber | Tro (2) | purple | 40 | CAM |
| ***Vriesea bituminosa* Wawra** | **Till.** | **Ber** | **Chi (9)** | **matt-white** | **55** | **C3** |
| *Vriesea bleheri* Roeth & W. Weber | Till. | Gö | Tro (2) | yellow | 55 | C3 |
| *Vriesea drepanocarpa* (Baker) Mez | Till. | Gö | Tro (8) | yellow | 40 | C3 |
| *Vriesea dubia* (L.B.Sm.) L.B.Sm. | Till. | Gö | Tro (2) | white | 20 | C3 |
| *Vriesea eltoniana* E.Pereira & Ivo | Till. | Gö | Tro (2) | yellow | 30 | C3 |
| ***Vriesea fenestralis* Linden & Andre** | **Till.** | **Gö** | **Chi (5)** | **matt-white** | **40** | **C3-CAM** |
| *Vriesea friburgensis tucumanensis* (Mez) L.B.Sm. | Till. | Gö | Tro (5) | yellow | 35 | C3 |
| *Vriesea guttata* Linden & Andre | Till. | Wup | Tro (2) | yellow | 25 | C3 |
| *Vriesea maxoniana* (L.B.Sm.) L.B.Sm. | Till. | Ber | Tro (15) | yellow | 50 | C3 |
| ***Vriesea nanuzae* Leme** | **Till.** | **Heid** | **Chi (23)** | **matt-white** | **30** | **C3** |
| ***Vriesea racinae* L.B.Sm.** | **Till.** | **Ber** | **Chi (2)** | **matt-white** | **40** | **C3** |
| *Vriesea saundersii* (Carriere) E.Morren | Till. | Heid | Tro (2) | yellow | 50 | C3 |
| *Vriesea scalaris* E.Morren | Till. | Ber | Tro (9) | yellow | 55 | C3-CAM |
| ***Vriesea unilateralis* (Baker) Mez** | **Till.** | **Gö** | **Chi (9)** | **matt-white** | **30** | **C3** |
| *Wallisia cyanea* Barfuss & W.Till | Till. | Gö | Psy (2) | purple | 55 | C3 |
| *Wallisia lindeniana* (Regel) E.Morren | Till. | Gö | Psy (2) | blue | 45 | C3 |
| *Wallisia pretiosa* (Mez) Barfuss & W.Till | Till. | Gö | Psy (2) | purple | 52 | C3 |
| ***Werauhia gladioliflora* (H.Wendland) J.R.Grant** | **Till.** | **Gö** | **Chi (15)** | **matt-white** | **40** | **C3** |
| ***Werauhia nutans* (L.B.Sm.) J.R.Grant** | **Till.** | **Gö** | **Chi (9)** | **matt-white** | **35** | **C3** |
| *Werauhia patzeltii* (Rauh) J.R.Grant | Till. | Gö | Tro (2) | white | 15 | C3 |
| ***Werauhia pectinata* (L.B.Sm.) J.R.Grant** | **Till.** | **Gö** | **Chi (2)** | **matt-white** | **35** | **C3** |
| ***Werauhia sanguinolenta* (Linden ex Cogniaux & Marchal) J.R.Grant** | **Till.** | **Gö** | **Chi (9)** | **matt-white** | **30** | **C3** |
| ***Werauhia werckleana* (Mez) J.R.Grant** | **Till.** | **Gö** | **Chi (2)** | **matt-white** | **40** | **C3** |

Subfamily: Brom. = Bromelioideae, Till. = Tillandsioideae, Pitc. = Pitcairnioideae. Botanical garden/University: Ber = Berlin (Germany), Boc = Bochum (Germany), Gö = Göttingen (Germany), Heid = Heidelberg (Germany), Wien (Austria), Wup = Wuppertal (Germany). Pollination type: Chi = chiropterophilous, Sphi = sphingophilous, Tro = trochilophilous, Ent = entomophilous, Psy = psychophilous. Bold type = bat-pollinated bromeliads.

References

1. Crayn, D.M., Winter, K., Schulte, K., Smith, J.A. (2015). Photosynthetic pathways in Bromeliaceae: Phylogenetic and ecological significance of CAM and C 3 based on carbon isotope ratios for 1893 species. Bot J Linn Soc 178(2):169–221. doi:10.1111/boj.12275

2. classified via flower morphology and pollination syndrome.

3. Zanata, T.B. (2014). Macroecologia das interações entre plantas e aves nectarívoras. PhD thesis. Universidade Federal do Paraná.

4. Dias, L.C.D. (2014). Biologia reprodutiva de *Aechmea bruggeri* Leme (Bromeliaceae): uma espécie endêmica da Floresta Atlântica ameaçada de extinção. PhD thesis. Universidade Federal de Juiz de Fora.

5. Martín González, A.M., Dalsgaard, B., Nogués-Bravo, D., Graham, C.H., Schleuning, M., Maruyama, P.K., et al. (2015). The macroecology of phylogenetically structured hummingbird-plant networks. Global Ecology and Biogeography 24(11):1212–24. doi:10.1111/geb.12355

6. Pierce, S., Gottsberger, R. (2001). Observations of hummingbird visits to bromeliads at the Cerro Jefe cloud forest, Panama. Journal of the Bromeliad Society: 25–34.

7. Benzing, D.H. (2000). Bromeliaceae: Profile of an adaptive radiation. Cambridge: Cambridge University Press. doi: 10.1017/CBO9780511565175

8. Tavares, D.C., Freitas, L., Gaglianone, M.C. (2016). Data compilation of hummingbird-pollinated plant species in the Brazilian Atlantic rain forest. Journal of Tropical Ecology. 32(04):335-339. doi:10.1594/PANGAEA.859056

9. Krömer, T., Kessler, M., Lohaus, G., Schmidt-Lebuhn, A.N. (2008). Nectar sugar composition and concentration in relation to pollination syndromes in Bromeliaceae. Plant Biol (Stuttg) 10(4):502–11. doi:10.1111/j.1438-8677.2008.00058.x

10. Santos, V.L., Versieux, L.M., Wanderley, M.D., Da Luz, C.F. (2017). Pollen morphology of *Alcantarea* giant bromeliads (Bromeliaceae, Tillandsioideae). Grana 57(1-2):117–36. doi:10.1080/00173134.2017.1310920

11. Martinelli, G. (1995). Reproductive biology of Bromeliaceae in the Atlantic rainforest of southeastern Brazil. PhD thesis. University of St Andrews.

12. Rodrigues, L.C., Rodrigues, M. (2014). Flowers visited by hummingbirds in the open habitats of the southeastern Brazilian mountaintops: Species composition and seasonality. Braz J Biol 74(3):659–76. doi:10.1590/bjb.2014.0097

13. Schütz, N. (2012). Systematics and evolution of the genus *Deuterocohnia* Mez (Bromeliaceae). PhD thesis. Universität Kassel.

14. Krapp, F., Barros Pinangé, D.S. de, Benko-Iseppon, A.M., Leme, E.M., Weising, K. (2014). Phylogeny and evolution of *Dyckia* (Bromeliaceae) inferred from chloroplast and nuclear sequences. Plant Syst Evol 100(17):597. doi:10.1007/s00606-014-0985-0

15. Krömer, T., Kessler, M., Herzog, S.K. (2006). Distribution and flowering ecology of bromeliads along two climatically contrasting elevational transects in the Bolivian Andes. Biotropica 38(2):183–95. doi:10.1111/j.1744-7429.2006.00124.x

16. Pierce, S., Winter, K., Griffiths, H. (2002). The role of CAM in high rainfall cloud forests: An *in situ* comparison of photosynthetic pathways in Bromeliaceae. Plant Cell Environ 25(9):1181–9. doi:10.1046/j.1365-3040.2002.00900.x

17. Mosti, S., Ross Friedman, C., Pacini, E., Brighigna, L., Papini, A. (2013). Nectary ultrastructure and secretory modes in three species of *Tillandsia* (Bromeliaceae) that have different pollinators. Botany 91(11):786–98. doi:10.1139/cjb-2013-0126

18. Aguilar-Rodríguez, P.A., MacSwiney G, M.C., Krömer, T., García-Franco, J.G., Knauer, A., Kessler, M. (2014). First record of bat-pollination in the species-rich genus *Tillandsia* (Bromeliaceae). Annals of Botany 113(6):1047–55. doi:10.1093/aob/mcu031

19. Fonseca, L.C., Vizentin-Bugoni, J., Rech, A.R., Alves, M.A. (2015). Plant-hummingbird interactions and temporal nectar availability in a restinga from Brazil. An Acad Bras Cienc 87(4):2163–75. doi:10.1590/0001-3765201520140349

20. González-Astorga, J., Cruz-Angón, A., Flores-Palacios, A., Vovides, A.P. (2004). Diversity and genetic structure of the Mexican endemic epiphyte *Tillandsia achyrostachys* E. Morr. ex Baker var. *achyrostachys* (Bromeliaceae). Annals of Botany 94(4):545–51. doi:10.1093/aob/mch171

21. Maruyama, P.K. (2015). Plant-hummingbird interactions = natural history and ecological networks = Interação entre plantas e beija-flores: História natural e redes ecológicas. PhD thesis. Universidade Estadual de Campinas.

22. Aguilar-Rodríguez, P.A., Krömer, T., García-Franco, J.G., MacSwiney G, M.C. (2016). From dusk till dawn: nocturnal and diurnal pollination in the epiphyte *Tillandsia heterophylla* (Bromeliaceae). Plant Biol (Stuttg) 18(1):37–45. doi:10.1111/plb.12319

23. Elton, M.C. (1997). Revision of the lithophytic *Vriesea* species from Minas Gerais State, Brazil: Part II. Journal of the Bromeliad Society (47):168–77.
