# Supplementary material for: What Do Nectarivorous Bats Like? Nectar Composition in Bromeliaceae With Special Emphasis on Bat-Pollinated Species
Source: Front Plant Sci. 2019 Feb 21;10:205. doi: 10.3389/fpls.2019.00205 (PMC6393375; doi:10.3389/fpls.2019.00205)
Supplement: Supplementary file 2 [file Table_2.docx]

Supplementary Material

What do nectarivorous bats like? Nectar composition in Bromeliaceae with special emphasis on bat-pollinated species

**Author: Thomas Göttlinger, Michael Schwerdtfeger, Kira Tiedge, Gertrud Lohaus***

***Correspondence:** Gertrud Lohaus (lohaus@uni-wuppertal.de)

Supplementary Table S2: Concentrations of the main three sugars in nectar of different Bromeliaceae species.

| **Species** | **Sum sugar [mM]** | **Percentages of sugars [%]** | | | **Sucrose-to-hexoses ratio** |
| --- | --- | --- | --- | --- | --- |
|  |  | **Glucose** | **Fructose** | **Sucrose** |  |
| *Aechmea abbreviata* | 1025 ± 312 | 24 | 22 | 54 | 1.3 ± 0.5 |
| *A. aquilega* | 1312 ± 108 | 19 | 20 | 61 | 1.6 ± 0.2 |
| *A. bruggeri* | 1290 ± 54 | 14 | 15 | 71 | 2.5 ± 0.1 |
| *A. cylindrata* | 1122 ± 209 | 19 | 13 | 68 | 2.4 ± 1.1 |
| *A. distichantha* | 1170 ± 90 | 17 | 14 | 69 | 2.3 ±0.4 |
| *A. eurycorymbus* | 1803 ± 782 | 10 | 8 | 82 | 5.2 ±2.5 |
| *A. fasciata* | 518 ± 13 | 21 | 21 | 58 | 1.4 ± 0.2 |
| *A.* *gamosepala* | 1127 ± 96 | 11 | 11 | 78 | 3.4 ± 0.1 |
| *A. gracilis* | 641 ± 57 | 12 | 18 | 70 | 2.3 ± 0.1 |
| *A. leptantha* | 1131 ± 27 | 21 | 22 | 57 | 1.3 ± 0.0 |
| *A. marauensis* | 1013 ± 277 | 23 | 26 | 51 | 1.1 ± 0.3 |
| *A. miniata discolor* | 1016 ± 23 | 15 | 17 | 68 | 2.1 ± 0.3 |
| *A. nudicaulis* | 620 ± 147 | 24 | 20 | 56 | 1.3 ± 0.1 |
| *A. penduliflora* | 1001 ± 58 | 15 | 19 | 66 | 2.0 ± 0.3 |
| *A. pyramidalis* | 1559 ± 142 | 11 | 7 | 82 | 4.6 ± 0.8 |
| *A. racinae* | 1177 ± 127 | 20 | 18 | 62 | 1.6 ± 0.3 |
| *A. recurvata* | 746 ± 73 | 21 | 13 | 66 | 2.0 ± 0.5 |
| *A. weilbachii* | 859 ± 75 | 19 | 20 | 61 | 1.6 ± 0.0 |
| *Alcantarea geniculata* | 978 ± 141 | 11 | 23 | 66 | 1.9 ± 0.2 |
| ***Alc. imperialis*** | **683 ± 226** | **31** | **31** | **38** | **0.6 ± 0.1** |
| *Billbergia amoena* | 430 ± 113 | 13 | 9 | 78 | 3.5 ± 0.3 |
| *B. brasiliensis* | 1011 ± 497 | 19 | 16 | 65 | 1.9 ± 0.7 |
| *B. buchholtzii* | 2289 ± 119 | 10 | 15 | 75 | 3.0 ± 0.7 |
| *B. distachia* | 1692 ± 598 | 12 | 18 | 70 | 2.3 ± 0.3 |
| *B. euphemiae* | 2022 ± 904 | 10 | 18 | 72 | 2.6 ± 0.3 |
| *B. fosteriana* | 1180 ± 189 | 16 | 16 | 68 | 2.2 ± 0.6 |
| *B. morelii* | 641 ± 55 | 15 | 15 | 70 | 2.4 ± 0.2 |
| *B. nutans* | 862 ± 39 | 8 | 9 | 83 | 4.8 ± 0.3 |
| *B. pyramidalis* | 1621 ± 168 | 17 | 18 | 65 | 1.9 ± 0.0 |
| *B. reichardtii* | 1396 ± 472 | 9 | 15 | 76 | 3.2 ± 0.2 |
| *B. viridiflora* | 1563 ± 393 | 21 | 20 | 59 | 1.5 ± 0.2 |
| *B. vittata* | 839 ± 181 | 8 | 9 | 83 | 4.8 ± 0.1 |
| *Deuterocohnia brevispicata* | 1444 ± 241 | 15 | 14 | 71 | 2.5 ± 0.4 |
| *Deu. longipetala* | 406 ± 235 | 9 | 9 | 82 | 5.5 ± 3.4 |
| *Deu. meziana subsp. carmineoviridiflora* | 504 ± 155 | 14 | 14 | 72 | 2.6 ± 0.1 |
| *Deu. recurvipetala* | 1020 ± 107 | 12 | 13 | 75 | 3.0 ± 0.1 |
| *Dyckia choristaminea* | 725 ± 286 | 20 | 14 | 66 | 2.0 ± 0.9 |
| *D. goehringii* | 736 ± 62 | 9 | 7 | 84 | 5.5 ± 0.5 |
| *D. leptostachya* | 1200 ± 38 | 21 | 17 | 62 | 1.7 ± 0.6 |
| *D. vestita* | 676 ± 272 | 14 | 11 | 75 | 2.9 ± 0.3 |
| *Guzmania acorifolia* | 296 ± 230 | 40 | 11 | 49 | 1.0 ± 0.2 |
| ***G. calothyrsus*** | **771 ± 33** | **29** | **30** | **41** | **0.7 ± 0.1** |
| *G. conifera* | 611 ± 25 | 15 | 18 | 67 | 2.1 ± 0.2 |
| ***G. cylindrica*** | **571 ± 52** | **41** | **33** | **26** | **0.4 ± 0.1** |
| ***G. farciminiformis*** | **583 ± 31** | **37** | **34** | **29** | **0.4 ± 0.1** |
| ***G. killipiana*** | **794 ± 56** | **39** | **35** | **26** | **0.4 ± 0.2** |
| *G. lingulata* | 667 ± 247 | 8 | 9 | 83 | 5.1 ± 1.3 |
| *G. melinonis* | 1048 ± 24 | 19 | 21 | 60 | 1.5 ± 0.2 |
| *G. monostachia* | 903 ± 61 | 17 | 18 | 65 | 1.8 ± 0.1 |
| *G. osyana* | 1409 ± 110 | 23 | 27 | 50 | 1.1 ± 0.4 |
| *G. rhonhofiana* | 248 ± 16 | 24 | 19 | 57 | 1.4 ± 0.4 |
| *G. roseiflora* | 772 ± 220 | 26 | 18 | 56 | 1.3 ± 0.5 |
| *G. sanguinea* | 996 ± 405 | 22 | 19 | 59 | 1.6 ± 0.6 |
| *G. variegata* | 513 ± 106 | 21 | 19 | 60 | 1.6 ± 0.4 |
| *G. wittmackii* | 673 ± 311 | 12 | 11 | 77 | 3.4 ± 0.5 |
| *G. zahnii* | 439 ± 39 | 31 | 22 | 47 | 0.9 ± 0.2 |
| *Hohenbergia correia-araujoi* | 1343 ± 147 | 21 | 17 | 62 | 1.7 ± 0.4 |
| *H. leopoldo-horstii* | 1598 ± 626 | 22 | 15 | 63 | 1.8 ± 0.6 |
| *H. rosea* | 1680 ± 200 | 27 | 25 | 48 | 0.9 ± 0.0 |
| *H. stellata* | 694 ± 183 | 27 | 19 | 54 | 1.2 ± 0.4 |
| *H. utriculosa* | 2147 ± 847 | 29 | 21 | 50 | 1.0 ± 0.2 |
| *Lemeltonia narthecioides* | 378 ± 174 | 16 | 18 | 66 | 2.0 ± 0.3 |
| *L. scaligera* | 894 ± 288 | 18 | 15 | 67 | 2.0 ± 0.2 |
| *Neoregelia ampullacea* | 1431 ± 161 | 26 | 21 | 53 | 1.1 ± 0.1 |
| *N. carolinae* | 1176 ± 194 | 24 | 20 | 56 | 1.3 ± 0.1 |
| *N. compacta* | 1153 ± 245 | 12 | 13 | 75 | 3.0 ± 0.4 |
| *N. farinosa* | 1203 ± 426 | 18 | 14 | 68 | 2.1 ± 0.2 |
| *N. fosteriana* | 2064 ± 342 | 18 | 18 | 64 | 1.8 ± 0.1 |
| *N. johannis* | 1197 ± 78 | 15 | 14 | 71 | 2.4 ± 0.1 |
| *N. kautskyi* | 928 ± 172 | 21 | 21 | 58 | 1.4 ± 0.2 |
| *N. laevis* | 1669 ± 166 | 19 | 16 | 65 | 1.9 ± 0.0 |
| *N. martinellii* | 1034 ± 86 | 23 | 19 | 58 | 1.4 ± 0.2 |
| *N. olens* | 999 ± 60 | 27 | 19 | 54 | 1.2 ± 0.2 |
| *N. pineliana* | 1327 ± 566 | 17 | 15 | 68 | 2.2 ± 0.2 |
| *N. seideliana* | 1285 ± 85 | 24 | 16 | 60 | 1.5 ± 0.0 |
| *N. wilsoniana* | 2005 ± 660 | 25 | 25 | 50 | 1.0 ± 0.2 |
| *Nidularium amazonicum* | 1581 ± 86 | 14 | 15 | 71 | 2.5 ± 0.1 |
| *Nid. innocentii* | 917 ± 247 | 24 | 19 | 57 | 1.4 ± 0.3 |
| *Nid. procerum* | 1125 ± 104 | 11 | 13 | 76 | 3.2 ± 0.4 |
| *Nid. purpureum* | 1112 ± 168 | 13 | 14 | 73 | 2.7 ± 0.2 |
| *Nid. rutilans* | 768 ± 40 | 19 | 19 | 62 | 1.6 ± 0.2 |
| *Nid. scheremetiewii* | 2553 ± 629 | 15 | 17 | 68 | 2.1 ± 0.1 |
| *Nid. utriculosum* | 899 ± 13 | 20 | 20 | 60 | 1.5 ± 0.2 |
| *Pitcairnia bromeliifolia* | 981 ± 294 | 16 | 9 | 75 | 3.1 ± 0.6 |
| *Pit. chiapensis* | 397 ± 6 | 13 | 10 | 77 | 3.5 ± 0.9 |
| *Pit. chiriquensis* | 923 ± 204 | 19 | 12 | 69 | 2.4 ± 1.2 |
| *Pit. grafii* | 1170 ± 235 | 22 | 21 | 57 | 1.3 ± 0.1 |
| ***Pit. recurvata*** | **715 ± 282** | **40** | **27** | **33** | **0.5 ± 0.1** |
| *Pit. rubronigriflora* | 751 ± 81 | 25 | 23 | 52 | 1.1 ± 0.1 |
| *Pit. sprucei* | 832 ± 146 | 13 | 14 | 73 | 2.7 ± 0.5 |
| *Pit. suaveolens* | 693 ± 73 | 28 | 23 | 49 | 1.0 ± 0.1 |
| *Pit. utcubambensis* | 484 ± 62 | 22 | 18 | 60 | 1.5 ± 0.1 |
| *Pit. xanthocalyx* | 731 ± 43 | 25 | 23 | 52 | 1.1 ± 0.1 |
| ***Pseudalcantarea grandis*** | **844 ± 304** | **34** | **35** | **31** | **0.5 ± 0.1** |
| ***Pse. macropetala*** | **1081 ± 59** | **32** | **31** | **37** | **0.6 ± 0.1** |
| ***Pse. viridiflora*** | **732 ± 284** | **39** | **26** | **35** | **0.5 ± 0.1** |
| *Puya coerulea var. violacea* | 1027 ± 67 | 25 | 19 | 56 | 1.3 ± 0.0 |
| *P. densiflora* | 991 ± 252 | 16 | 14 | 70 | 2.3 ± 0.4 |
| ***P. ferruginea*** | **1007 ± 413** | **42** | **34** | **24** | **0.3 ± 0.1** |
| *P. spathacea* | 1204 ± 95 | 15 | 17 | 68 | 2.1 ± 0.0 |
| *Quesnelia edmundoi* | 1121 ± 797 | 30 | 24 | 46 | 0.9 ± 0.2 |
| *Q. lateralis* | 1125 ± 322 | 15 | 13 | 72 | 2.6 ± 0.6 |
| *Q. quesneliana* | 1096 ± 137 | 20 | 20 | 60 | 1.5 ± 0.0 |
| *Tillandsia achyrostachys* | 1148 ± 215 | 18 | 12 | 70 | 2.4 ± 0.4 |
| *T. aeranthos* | 1150 ± 286 | 22 | 18 | 60 | 1.5 ± 0.0 |
| *T. caput-medusae* | 816 ± 68 | 10 | 12 | 78 | 3.6 ± 0.2 |
| *T. circinnatoides* | 329 ± 145 | 8 | 12 | 80 | 4.0 ± 0.1 |
| *T. clavigera* | 455 ± 63 | 33 | 16 | 51 | 1.1 ± 0.2 |
| *T. concolor* | 551 ± 272 | 9 | 18 | 73 | 2.8 ± 0.4 |
| *T. flabellata* | 950 ± 24 | 22 | 20 | 58 | 1.4 ± 0.2 |
| *T. foliosa* | 1217 ± 261 | 10 | 13 | 77 | 3.5 ± 0.6 |
| *T. funckiana* | 389 ± 136 | 6 | 9 | 85 | 6.1 ± 1.8 |
| *T. gerdae* | 486 ± 87 | 7 | 5 | 88 | 8.1 ± 2.6 |
| ***T. heterophylla*** | **978 ± 75** | **38** | **39** | **23** | **0.3 ± 0.0** |
| *T. ionantha* | 483 ± 91 | 6 | 9 | 75 | 6.4 ± 2.3 |
| *T. ixioides* | 1271 ± 42 | 12 | 13 | 75 | 3.0 ± 0.3 |
| *T. makoyana* | 1658 ± 312 | 5 | 9 | 86 | 6.0 ± 0.6 |
| *T. malzinei* | 652 ± 61 | 10 | 16 | 74 | 2.9 ± 0.4 |
| *T. polystachia* | 554 ± 128 | 20 | 15 | 65 | 1.9 ± 0.4 |
| *T. ponderosa* | 736 ± 20 | 16 | 15 | 69 | 2.2 ± 0.1 |
| *T. propagulifera* | 232 ± 51 | 13 | 15 | 72 | 2.6 ± 0.2 |
| ***T. rauhii*** | **496 ± 72** | **41** | **30** | **29** | **0.4 ± 0.1** |
| *T. roland-gosselinii* | 1042 ± 53 | 8 | 7 | 85 | 5.6 ± 0.4 |
| *T. tricolor* | 824 ± 211 | 7 | 7 | 86 | 6.1 ± 0.5 |
| ***Vriesea bituminosa*** | **1477 ± 291** | **33** | **31** | **36** | **0.6 ± 0.0** |
| *V. bleheri* | 703 ± 117 | 16 | 17 | 67 | 2.0 ± 0.4 |
| *V. drepanocarpa* | 1082 ± 479 | 11 | 14 | 75 | 3.3 ± 1.0 |
| *V. dubia* | 337 ± 124 | 18 | 19 | 63 | 2.0 ± 1.3 |
| *V. eltoniana* | 738 ± 82 | 19 | 15 | 66 | 2.2 ± 1.1 |
| ***V. fenestralis*** | **961 ± 155** | **37** | **37** | **26** | **0.4 ± 0.1** |
| *V. friburgensis tucumanensis* | 1300 ± 80 | 17 | 17 | 66 | 2.0 ± 0.1 |
| *V. guttata* | 2249 ± 408 | 18 | 13 | 69 | 2.2 ± 0.4 |
| *V. maxoniana* | 353 ± 53 | 9 | 7 | 84 | 5.2 ± 0.6 |
| ***V. nanuzae*** | **819 ± 402** | **33** | **26** | **41** | **0.7 ± 0.0** |
| ***V. racinae*** | **942 ± 192** | **40** | **32** | **28** | **0.4 ± 0.2** |
| *V. saundersii* | 530 ± 75 | 18 | 8 | 74 | 3.1 ± 1.0 |
| *V. scalaris* | 966 ± 108 | 7 | 8 | 85 | 5.4 ± 0.1 |
| ***V. unilateralis*** | **509 ± 84** | **41** | **33** | **26** | **0.4 ± 0.2** |
| *Wallisia cyanea* | 606 ± 266 | 18 | 16 | 66 | 2.0 ± 0.8 |
| *W. lindeniana* | 944 ± 384 | 14 | 13 | 73 | 2.9 ± 1.2 |
| *W. pretiosa* | 321 ± 99 | 10 | 9 | 81 | 5.0 ± 2.7 |
| ***Werauhia gladioliflora*** | **1035 ± 109** | **34** | **32** | **34** | **0.5 ± 0.0** |
| ***Wer. nutans*** | **1024 ± 402** | **37** | **30** | **33** | **0.5 ± 0.2** |
| *Wer. patzeltii* | 861 ± 74 | 20 | 15 | 65 | 1.9 ± 0.3 |
| ***Wer. pectinata*** | **632 ± 36** | **50** | **35** | **15** | **0.2 ± 0.1** |
| ***Wer. sanguinolenta*** | **1057 ± 205** | **36** | **26** | **38** | **0.6 ± 0.1** |
| ***Wer. werckleana*** | **1232 ± 256** | **41** | **35** | **24** | **0.3 ± 0.0** |

Bold type = bat-pollinated bromeliads.
