# Supplementary material for: What Do Nectarivorous Bats Like? Nectar Composition in Bromeliaceae With Special Emphasis on Bat-Pollinated Species
Source: Front Plant Sci. 2019 Feb 21;10:205. doi: 10.3389/fpls.2019.00205 (PMC6393375; doi:10.3389/fpls.2019.00205)
Supplement: Supplementary file 3 [file Table_3.docx]

Supplementary Material

What do nectarivorous bats like? Nectar composition in Bromeliaceae with special emphasis on bat-pollinated species

**Author: Thomas Göttlinger, Michael Schwerdtfeger, Kira Tiedge, Gertrud Lohaus***

***Correspondence:** Gertrud Lohaus (lohaus@uni-wuppertal.de)

Supplementary Table S3: Amino acid concentration, proportions of the most abundant amino acids and the ratio of sugars to amino acids in nectar of different Bromeliaceae species.

| **Species** | **Sum amino** | **Percentages of amino acids [%]** | | | | **Sum sugars [mM]/Sum** |
| --- | --- | --- | --- | --- | --- | --- |
|  | **acids [mM]** | Asn & Gln | Pro | Essential | Residual | **amino acids [mM]** |
| *Aechmea abbreviata* | 3.6 ± 2.4 | 65 | 1 | 14 | 20 | 281 |
| *A. aquilega* | 2.0 ± 0.2 | 6 | 31 | 38 | 25 | 666 |
| *A. bruggeri* | 0.2 ± 0.0 | 16 | 14 | 23 | 47 | 6589 |
| *A. cylindrata* | 0.3 ± 0.1 | 38 | 16 | 22 | 24 | 4063 |
| *A. distichantha* | 0.7 ± 0.3 | 44 | 8 | 21 | 27 | 1690 |
| *A. eurycorymbus* | 1.7 ± 1.2 | 41 | 1 | 30 | 28 | 1036 |
| *A. fasciata* | 0.5 ± 0.0 | 28 | 4 | 39 | 29 | 963 |
| *A.* *gamosepala* | 1.1 ± 0.1 | 64 | 15 | 8 | 13 | 1073 |
| *A. gracilis* | 2.1 ± 0.1 | 18 | 1 | 39 | 42 | 299 |
| *A. leptantha* | 0.9 ± 0.3 | 21 | 55 | 7 | 17 | 1198 |
| *A. marauensis* | 0.4 ± 0.2 | 12 | 4 | 31 | 53 | 2754 |
| *A. miniata discolor* | 1.7 ± 0.6 | 16 | 31 | 27 | 26 | 596 |
| *A. nudicaulis* | 0.4 ± 0.0 | 17 | 23 | 26 | 34 | 1617 |
| *A. penduliflora* | 0.2 ± 0.1 | 22 | 14 | 28 | 36 | 4660 |
| *A. pyramidalis* | 1.3 ± 0.4 | 30 | 4 | 25 | 41 | 1221 |
| *A. racinae* | 0.4 ± 0.1 | 62 | 2 | 16 | 20 | 3108 |
| *A. recurvata* | 0.9 ± 0.0 | 47 | 1 | 26 | 26 | 859 |
| *A. weilbachii* | 0.8 ± 0.1 | 45 | 0 | 28 | 27 | 1107 |
| *Alcantarea geniculata* | 0.7 ± 0.3 | 73 | 1 | 5 | 21 | 1413 |
| ***Alc. imperialis*** | **1.0 ± 0.2** | **34** | **12** | **30** | **24** | **715** |
| *Billbergia amoena* | 0.1 ± 0.1 | 66 | 1 | 18 | 15 | 2975 |
| *B. brasiliensis* | 0.1 ± 0.0 | 15 | 53 | 11 | 21 | 8544 |
| *B. buchholtzii* | 0.2 ± 0.0 | 40 | 0 | 17 | 43 | 9658 |
| *B. distachia* | 0,7 ± 0,2 | 46 | 13 | 20 | 21 | 534 |
| *B. euphemiae* | 0.2 ± 0.1 | 54 | 17 | 12 | 17 | 10044 |
| *B. fosteriana* | 0.6 ± 0.3 | 46 | 0 | 19 | 35 | 1608 |
| *B. morelii* | 0.6 ± 0.1 | 37 | 17 | 16 | 30 | 1112 |
| *B. nutans* | 0.4 ± 0.1 | 26 | 51 | 10 | 13 | 2392 |
| *B. pyramidalis* | 0.1 ± 0.1 | 32 | 3 | 16 | 49 | 14629 |
| *B. reichardtii* | 0.1 ± 0.0 | 35 | 7 | 21 | 37 | 24579 |
| *B. viridiflora* | 0.8 ± 0.6 | 27 | 3 | 36 | 34 | 2010 |
| *B. vittata* | 0.1 ± 0.0 | 29 | 27 | 18 | 26 | 11551 |
| *Deuterocohnia brevispicata* | 1.6 ± 0.2 | 55 | 1 | 24 | 20 | 885 |
| *Deu. longipetala* | 3.8 ± 0.2 | 70 | 0 | 12 | 18 | 108 |
| *Deu. meziana subsp. carmineoviridiflora* | 0.2 ± 0.1 | 21 | 0 | 24 | 55 | 2978 |
| *Deu. recurvipetala* | 0.7 ± 0.3 | 83 | 2 | 7 | 8 | 1559 |
| *Dyckia choristaminea* | 7.1 ± 0.1 | 57 | 1 | 25 | 17 | 102 |
| *D. goehringii* | 1.2 ± 0.4 | 24 | 11 | 30 | 35 | 634 |
| *D. leptostachya* | 0.3 ± 0.2 | 34 | 26 | 11 | 29 | 3391 |
| *D. vestita* | 2.7 ± 1.0 | 53 | 4 | 20 | 23 | 217 |
| *Guzmania acorifolia* | 6.6 ± 1.1 | 34 | 0 | 40 | 26 | 45 |
| ***G. calothyrsus*** | **1.2 ± 0.2** | **41** | **2** | **36** | **21** | **665** |
| *G. conifera* | 1.6 ± 0.0 | 17 | 36 | 27 | 20 | 371 |
| ***G. cylindrica*** | **0.3 ± 0.1** | **28** | **2** | **46** | **24** | **1601** |
| ***G. farciminiformis*** | **2.1 ± 0.3** | **34** | **3** | **29** | **34** | **279** |
| ***G. killipiana*** | **7.3 ± 0.9** | **43** | **1** | **21** | **35** | **109** |
| *G. lingulata* | 7.8 ± 2.9 | 59 | 0 | 21 | 20 | 79 |
| *G. melinonis* | 3.1 ± 0.6 | 41 | 1 | 38 | 20 | 342 |
| *G. monostachia* | 0.1 ± 0.0 | 27 | 14 | 31 | 28 | 6512 |
| *G. osyana* | 2.3 ± 1.1 | 34 | 3 | 31 | 32 | 616 |
| *G. rhonhofiana* | 0.4 ± 0.5 | 26 | 28 | 25 | 21 | 563 |
| *G. roseiflora* | 1.9 ± 0.9 | 53 | 0 | 37 | 10 | 411 |
| *G. sanguinea* | 1.5 ± 1.6 | 44 | 1 | 24 | 31 | 674 |
| *G. variegata* | 1.0 ± 0.2 | 44 | 1 | 29 | 26 | 504 |
| *G. wittmackii* | 0.5 ± 0.1 | 13 | 6 | 35 | 46 | 1476 |
| *G. zahnii* | 2.1 ± 0.1 | 57 | 0 | 25 | 18 | 206 |
| *Hohenbergia correia-araujoi* | 0.2 ± 0.2 | 7 | 2 | 33 | 58 | 6148 |
| *H. leopoldo-horstii* | 0.6 ± 0.3 | 24 | 2 | 30 | 44 | 2815 |
| *H. rosea* | 0.2 ± 0.1 | 33 | 0 | 26 | 41 | 6945 |
| *H. stellata* | 1.4 ± 1.3 | 17 | 0 | 40 | 43 | 513 |
| *H. utriculosa* | 0.9 ± 0.5 | 40 | 0 | 27 | 33 | 2470 |
| *Lemeltonia narthecioides* | 5.3 ± 3.4 | 79 | 0 | 11 | 10 | 71 |
| *L. scaligera* | 0.3 ± 0.0 | 49 | 1 | 18 | 32 | 3519 |
| *Neoregelia ampullacea* | 3.7 ± 1.6 | 28 | 2 | 35 | 35 | 386 |
| *N. carolinae* | 1.9 ± 0.3 | 24 | 1 | 29 | 46 | 637 |
| *N. compacta* | 2.2 ± 0.3 | 23 | 0 | 35 | 42 | 537 |
| *N. farinosa* | 0.2 ± 0.1 | 40 | 6 | 35 | 19 | 7176 |
| *N. fosteriana* | 0.7 ± 0.1 | 19 | 3 | 32 | 46 | 3190 |
| *N. johannis* | 0.4 ± 0.1 | 15 | 0 | 44 | 41 | 2786 |
| *N. kautskyi* | 0.2 ± 0.1 | 33 | 8 | 25 | 34 | 4010 |
| *N. laevis* | 1.6 ± 1.1 | 15 | 2 | 39 | 44 | 880 |
| *N. martinellii* | 0.8 ± 0.4 | 21 | 3 | 22 | 54 | 1360 |
| *N. olens* | 6.7 ± 0.9 | 21 | 0 | 44 | 35 | 150 |
| *N. pineliana* | 0.5 ± 0.3 | 2 | 19 | 12 | 67 | 2465 |
| *N. seideliana* | 0.1 ± 0.0 | 23 | 1 | 44 | 32 | 12880 |
| *N. wilsoniana* | 0.3 ± 0.0 | 43 | 0 | 29 | 28 | 5891 |
| *Nidularium amazonicum* | 1.8 ± 0.2 | 58 | 0 | 15 | 27 | 893 |
| *Nid. innocentii* | 1.5 ± 0.4 | 23 | 0 | 31 | 46 | 614 |
| *Nid. procerum* | 2.6 ± 0.9 | 59 | 0 | 15 | 26 | 441 |
| *Nid. purpureum* | 2.4 ± 0.9 | 13 | 0 | 30 | 57 | 466 |
| *Nid. rutilans* | 0.3 ± 0.0 | 30 | 0 | 42 | 28 | 2632 |
| *Nid. scheremetiewii* | 2.4 ± 0.7 | 39 | 0 | 28 | 33 | 1063 |
| *Nid. utriculosum* | 0.4 ± 0.0 | 36 | 2 | 32 | 30 | 2024 |
| *Pitcairnia bromeliifolia* | 16.2 ± 2.5 | 40 | 1 | 21 | 38 | 47 |
| *Pit. chiapensis* | 8.2 ± 0.5 | 11 | 0 | 32 | 57 | 49 |
| *Pit. chiriquensis* | 2.5 ± 0.6 | 24 | 1 | 24 | 51 | 371 |
| *Pit. grafii* | 12.6 ± 2.8 | 30 | 0 | 12 | 58 | 64 |
| ***Pit. recurvata*** | **0.3 ± 0.0** | **38** | **5** | **29** | **28** | **2533** |
| *Pit. rubronigriflora* | 6.6 ± 1.5 | 47 | 1 | 9 | 43 | 114 |
| *Pit. sprucei* | 13.0 ± 3.9 | 46 | 1 | 14 | 39 | 47 |
| *Pit. suaveolens* | 0.8 ± 0.2 | 32 | 5 | 31 | 32 | 909 |
| *Pit. utcubambensis* | 11.3 ± 1.7 | 20 | 0 | 35 | 45 | 39 |
| *Pit. xanthocalyx* | 0.9 ± 0.0 | 33 | 1 | 24 | 42 | 819 |
| ***Pseudalcantarea grandis*** | **3.9 ± 1.4** | **32** | **0** | **18** | **50** | **219** |
| ***Pse. macropetala*** | **1.6 ± 0.3** | **22** | **1** | **34** | **43** | **678** |
| ***Pse. viridiflora*** | **0.7 ± 0.1** | **20** | **2** | **17** | **61** | **1002** |
| *Puya coerulea var. violacea* | 3.9 ± 1.0 | 24 | 0 | 35 | 41 | 261 |
| *P. densiflora* | 0.3 ± 0.0 | 24 | 1 | 44 | 31 | 3883 |
| ***P. ferruginea*** | **3,9 ± 0.9** | **15** | **57** | **15** | **13** | **256** |
| *P. spathacea* | 0.7 ± 0.2 | 26 | 0 | 48 | 26 | 1734 |
| *Quesnelia edmundoi* | 1.4 ± 0.3 | 41 | 1 | 29 | 29 | 776 |
| *Q. lateralis* | 0.4 ± 0.2 | 82 | 3 | 8 | 7 | 2647 |
| *Q. quesneliana* | 1.4 ± 0.3 | 24 | 2 | 26 | 48 | 799 |
| *Tillandsia achyrostachys* | 0.2 ± 0.0 | 56 | 2 | 18 | 24 | 7314 |
| *T. aeranthos* | 1.4 ± 0.1 | 30 | 1 | 29 | 40 | 814 |
| *T. caput-medusae* | 0.4 ± 0.1 | 43 | 0 | 26 | 31 | 2038 |
| *T. circinnatoides* | 0.3 ± 0.1 | 36 | 3 | 41 | 20 | 949 |
| *T. clavigera* | 0.7 ± 0.5 | 22 | 6 | 38 | 34 | 643 |
| *T. concolor* | 0.2 ± 0.1 | 47 | 3 | 7 | 43 | 2827 |
| *T. flabellata* | 1.3 ± 0.4 | 61 | 5 | 18 | 16 | 749 |
| *T. foliosa* | 0.5 ± 0.1 | 31 | 12 | 18 | 39 | 2670 |
| *T. funckiana* | 2.0 ± 1.0 | 40 | 1 | 37 | 22 | 197 |
| *T. gerdae* | 1.4 ± 0.5 | 61 | 1 | 13 | 25 | 341 |
| ***T. heterophylla*** | **0.3 ± 0.0** | **41** | **2** | **25** | **32** | **3048** |
| *T. ionantha* | 0.6 ± 0.0 | 29 | 7 | 11 | 53 | 800 |
| *T. ixioides* | 1.4 ± 0.8 | 48 | 1 | 33 | 18 | 928 |
| *T. makoyana* | 0.2 ± 0.0 | 20 | 5 | 34 | 41 | 7260 |
| *T. malzinei* | 0.5 ± 0.1 | 5 | 0 | 56 | 39 | 1251 |
| *T. polystachia* | 0.1 ± 0.1 | 4 | 61 | 10 | 25 | 4390 |
| *T. ponderosa* | 0.1 ± 0.0 | 30 | 12 | 4 | 54 | 7907 |
| *T. propagulifera* | 0.2 ± 0.1 | 27 | 26 | 21 | 26 | 1083 |
| ***T. rauhii*** | **7.4 ± 0.3** | **59** | **0** | **24** | **17** | **68** |
| *T. roland-gosselinii* | 0.2 ± 0.0 | 25 | 4 | 39 | 32 | 5909 |
| *T. tricolor* | 1.3 ± 0.5 | 36 | 0 | 26 | 38 | 620 |
| ***Vriesea bituminosa*** | **2.8 ± 0.2** | **46** | **14** | **20** | **20** | **525** |
| *V. bleheri* | 0.1 ± 0.0 | 32 | 20 | 12 | 36 | 13338 |
| *V. drepanocarpa* | 0.2 ± 0.1 | 30 | 7 | 7 | 56 | 5516 |
| *V. dubia* | 3.6 ± 0.1 | 60 | 0 | 24 | 16 | 94 |
| *V. eltoniana* | 0.1 ± 0.0 | 43 | 1 | 20 | 36 | 7994 |
| ***V. fenestralis*** | **1.2 ± 0.4** | **22** | **8** | **47** | **23** | **863** |
| *V. friburgensis tucumanensis* | 1.4 ± 0.1 | 40 | 0 | 31 | 29 | 919 |
| *V. guttata* | 0.6 ± 0.1 | 36 | 1 | 20 | 43 | 4126 |
| *V. maxoniana* | 0.6 ± 0.3 | 16 | 0 | 39 | 45 | 574 |
| ***V. nanuzae*** | **0.5 ± 0.1** | **1** | **2** | **62** | **35** | **1666** |
| ***V. racinae*** | **1.6 ± 0.6** | **23** | **51** | **13** | **13** | **587** |
| *V. saundersii* | 0.3 ± 0.1 | 51 | 10 | 10 | 29 | 1941 |
| *V. scalaris* | 0.2 ± 0.2 | 19 | 9 | 20 | 52 | 3888 |
| ***V. unilateralis*** | **3.5 ± 0.3** | **15** | **27** | **33** | **25** | **144** |
| *Wallisia cyanea* | 0.1 ± 0.0 | 38 | 3 | 22 | 37 | 4264 |
| *W. lindeniana* | 0.7 ± 0.3 | 51 | 1 | 28 | 20 | 1439 |
| *W. pretiosa* | 0.2 ± 0.1 | 46 | 3 | 19 | 32 | 1696 |
| ***Werauhia gladioliflora*** | **2.9 ± 0.8** | **36** | **37** | **10** | **17** | **362** |
| ***Wer. nutans*** | **3.6 ± 1.0** | **50** | **5** | **33** | **12** | **283** |
| *Wer. patzeltii* | 1.6 ± 0.1 | 53 | 1 | 34 | 12 | 552 |
| ***Wer. pectinata*** | **0.2 ± 0.1** | **5** | **3** | **70** | **22** | **2402** |
| ***Wer. sanguinolenta*** | **2.9 ± 1.6** | **69** | **9** | **13** | **9** | **362** |
| ***Wer. werckleana*** | **5.1 ± 0.7** | **36** | **39** | **17** | **8** | **242** |

Bold type = bat-pollinated bromeliads. Essential amino acids = arg, his, ile, leu, lys, met, phe, thr, trp, val. Residual amino acids = ala, asp, gaba, glu, gly, ser, tyr.
