# Supplementary material for: What Do Nectarivorous Bats Like? Nectar Composition in Bromeliaceae With Special Emphasis on Bat-Pollinated Species
Source: Front Plant Sci. 2019 Feb 21;10:205. doi: 10.3389/fpls.2019.00205 (PMC6393375; doi:10.3389/fpls.2019.00205)
Supplement: Supplementary file 4 [file Table_4.docx]

Supplementary Material

What do nectarivorous bats like? Nectar composition in Bromeliaceae with special emphasis on bat-pollinated species

**Author: Thomas Göttlinger, Michael Schwerdtfeger, Kira Tiedge, Gertrud Lohaus***

***Correspondence:** Gertrud Lohaus (lohaus@uni-wuppertal.de)

Supplementary Table S4: Concentrations of inorganic cations, anions, malate, and the ion composition in nectar of different Bromeliaceae species.

| **Species** | **Sum cations** | **Percentages of cations [%]** | | | **Sum anions** | **Percentages of anions [%]** | | | **Malate [mM]** |
| --- | --- | --- | --- | --- | --- | --- | --- | --- | --- |
|  | **[mM]** | K^+^ | Na^+^ | Mg^2+^ & Ca^2+^ | **[mM]** | Cl^-^ | PO_4_^3-^ | SO_4_^2-^ |  |
| *Aechmea abbreviata* | 0.4 ± 0.0 | 28 | 52 | 20 | 1.4 ± 0.0 | 88 | 9 | 3 | 0.13 |
| *A. aquilega* | 1.0 ± 0.2 | 79 | 17 | 4 | 1.2 ± 0.0 | 75 | 19 | 6 | 0.05 |
| *A. bruggeri* | 0.5 ± 0.0 | 73 | 11 | 16 | 0.4 ± 0.1 | 100 | 0 | 0 | 0.00 |
| *A. cylindrata* | 1.0 ± 0.8 | 46 | 36 | 18 | 0.7 ± 0.1 | 75 | 15 | 10 | 0.01 |
| *A. distichantha* | 1.0 ± 0.2 | 58 | 23 | 19 | 0.5 ± 0.2 | 69 | 10 | 21 | 0.01 |
| *A. eurycorymbus* | 1.0 ± 0.5 | 76 | 8 | 16 | 1.1 ± 0.3 | 87 | 2 | 11 | 0.01 |
| *A. fasciata* | 0.8 ± 0.1 | 76 | 11 | 13 | 1.3 ± 0.1 | 81 | 18 | 1 | 0.00 |
| *A.* *gamosepala* | 0.5 ± 0.1 | 64 | 22 | 14 | 0.9 ± 0.1 | 81 | 6 | 13 | 0.00 |
| *A. gracilis* | 0.6 ± 0.3 | 50 | 22 | 28 | 0.8 ± 0.3 | 54 | 32 | 14 | 0.07 |
| *A. leptantha* | 1.2 ± 0.4 | 82 | 10 | 8 | 0.2 ± 0.0 | 49 | 42 | 9 | 0.05 |
| *A. marauensis* | 1.4 ± 0.0 | 87 | 5 | 8 | 0.3 ± 0.1 | 65 | 15 | 20 | 0.23 |
| *A. miniata discolor* | 2.9 ± 0.5 | 71 | 15 | 14 | 4.1 ± 0.6 | 96 | 3 | 1 | 0.37 |
| *A. nudicaulis* | 2.1 ± 1.3 | 61 | 24 | 15 | 0.9 ± 0.1 | 75 | 16 | 9 | 0.08 |
| *A. penduliflora* | 0.6 ± 0.4 | 60 | 20 | 20 | 0.3 ± 0.1 | 72 | 0 | 28 | 0.00 |
| *A. pyramidalis* | 1.5 ± 0.4 | 59 | 20 | 21 | n.d. | n.d. | n.d. | n.d. | 0.00 |
| *A. racinae* | 1.7 ± 0.6 | 66 | 27 | 7 | 0.9 ± 0.1 | 81 | 4 | 15 | 0.03 |
| *A. recurvata* | 0.5 ± 0.2 | 18 | 70 | 12 | 0.4 ± 0.3 | 82 | 0 | 18 | 0.00 |
| *A. weilbachii* | 0.6 ± 0.4 | 40 | 38 | 22 | 0.7 ± 0.1 | 73 | 22 | 5 | 0.07 |
| *Alcantarea geniculata* | 1.1 ± 0.1 | 75 | 20 | 5 | 0.3 ± 0.1 | 66 | 33 | 1 | 0.27 |
| ***Alc. imperialis*** | **1.9 ± 0.8** | **85** | **8** | **7** | **0.7 ± 0.6** | **74** | **13** | **13** | **0.53** |
| *Billbergia amoena* | 0.3 ± 0.1 | 26 | 64 | 10 | 0.7 ± 0.2 | 98 | 0 | 2 | 0.01 |
| *B. brasiliensis* | 1.1 ± 0.2 | 65 | 28 | 7 | 0.7 ± 0.2 | 52 | 29 | 19 | 0.00 |
| *B. buchholtzii* | 0.7 ± 0.3 | 84 | 8 | 8 | 0.8 ± 0.1 | 43 | 40 | 17 | 0.03 |
| *B. distachia* | 0.3 ± 0.2 | 12 | 55 | 33 | 0.7 ± 0.1 | 73 | 25 | 2 | 0.20 |
| *B. euphemiae* | 0.7 ± 0.2 | 56 | 31 | 13 | 0.4 ± 0.0 | 89 | 1 | 10 | 0.01 |
| *B. fosteriana* | 1.2 ± 0.7 | 70 | 26 | 4 | 1.0 ± 0.5 | 79 | 9 | 12 | 0.17 |
| *B. morelii* | 1.2 ± 0.0 | 93 | 3 | 4 | 1.0 ± 0.2 | 97 | 1 | 2 | 0.23 |
| *B. nutans* | 0.1 ± 0.0 | 78 | 22 | 0 | 0.7 ± 0.1 | 85 | 13 | 2 | 0.02 |
| *B. pyramidalis* | 0.1 ± 0.0 | 23 | 44 | 33 | n.d. | n.d. | n.d. | n.d. | 0.00 |
| *B. reichardtii* | 0.3 ± 0.2 | 45 | 51 | 4 | 0.1 ± 0.0 | 60 | 19 | 21 | 0.06 |
| *B. viridiflora* | 0.1 ± 0.1 | 70 | 30 | 0 | n.d. | n.d. | n.d. | n.d. | 0.00 |
| *B. vittata* | 0.3 ± 0.3 | 53 | 28 | 19 | 0.2 ± 0.0 | 90 | 5 | 5 | 0.04 |
| *Deuterocohnia brevispicata* | 0.8 ± 0.1 | 62 | 30 | 8 | 0.8 ± 0.1 | 68 | 20 | 12 | 0.09 |
| *Deu. longipetala* | 1.4 ± 0.3 | 42 | 28 | 30 | 0.8 ± 0.2 | 78 | 0 | 22 | 0.02 |
| *Deu. meziana subsp. carmineoviridiflora* | 0.2 ± 0.0 | 51 | 18 | 31 | 0.3 ± 0.0 | 100 | 0 | 0 | 0.00 |
| *Deu. recurvipetala* | 0.6 ± 0.1 | 71 | 26 | 3 | 0.9 ± 0.4 | 83 | 0 | 17 | 0.04 |
| *Dyckia choristaminea* | 1.0 ± 0.7 | 32 | 35 | 33 | 0.7 ± 0.4 | 81 | 8 | 11 | 0.04 |
| *D. goehringii* | 3.1 ± 0.7 | 89 | 7 | 4 | 0.9 ± 0.3 | 21 | 70 | 9 | 0.19 |
| *D. leptostachya* | 2.1 ± 1.5 | 83 | 14 | 3 | 0.5 ± 0.3 | 40 | 46 | 14 | 0.01 |
| *D. vestita* | 0.8 ± 0.5 | 59 | 29 | 12 | 0.3 ± 0.1 | 43 | 42 | 15 | 0.03 |
| *Guzmania acorifolia* | 12.4 ± 0.8 | 85 | 2 | 13 | 6.1 ± 1.2 | 83 | 12 | 5 | 0.14 |
| ***G. calothyrsus*** | **9.9 ± 3.5** | **98** | **1** | **1** | **9.8 ± 0.5** | **94** | **5** | **1** | **0.09** |
| *G. conifera* | 7.0 ± 1.8 | 76 | 22 | 2 | 16.9 ± 0.9 | 91 | 5 | 4 | 0.32 |
| ***G. cylindrica*** | **12.2 ± 1.9** | **95** | **4** | **1** | **5.5 ± 1.8** | **85** | **1** | **14** | **7.09** |
| ***G. farciminiformis*** | **6.3 ± 0.8** | **30** | **68** | **2** | **6.8 ± 2.5** | **80** | **4** | **16** | **0.08** |
| ***G. killipiana*** | **10.0 ± 1.1** | **95** | **2** | **3** | **11.9 ± 1.4** | **75** | **18** | **7** | **3.91** |
| *G. lingulata* | 3.5 ± 0.9 | 91 | 9 | 0 | 1.9 ± 0.5 | 84 | 11 | 5 | 0.39 |
| *G. melinonis* | 6.8 ± 1.4 | 94 | 2 | 4 | 7.7 ± 1.2 | 96 | 3 | 1 | 0.42 |
| *G. monostachia* | 2.0 ± 0.1 | 98 | 1 | 1 | 7.7 ± 0.9 | 96 | 1 | 3 | 0.18 |
| *G. osyana* | 7.6 ± 1.4 | 94 | 2 | 4 | 8.1 ± 2.5 | 86 | 8 | 6 | 0.52 |
| *G. rhonhofiana* | 3.8 ± 0.1 | 94 | 2 | 4 | 1.0 ± 0.5 | 100 | 0 | 0 | 0.17 |
| *G. roseiflora* | 12.3 ± 1.1 | 93 | 3 | 4 | 5.5 ± 2.2 | 96 | 0 | 4 | 0.49 |
| *G. sanguinea* | 4.9 ± 0.8 | 86 | 4 | 10 | 4.7 ± 1.0 | 82 | 12 | 6 | 0.33 |
| *G. variegata* | 4.1 ± 2.2 | 92 | 5 | 3 | 3.9 ± 0.6 | 93 | 2 | 6 | 0.65 |
| *G. wittmackii* | 2.1 ± 0.2 | 11 | 24 | 65 | 0.5 ± 0.2 | 60 | 28 | 12 | 0.06 |
| *G. zahnii* | 2.9 ± 0.6 | 91 | 3 | 6 | 4.2 ± 0.7 | 96 | 2 | 2 | 0.32 |
| *Hohenbergia correia-araujoi* | 0.7 ± 0.7 | 52 | 32 | 16 | 0.2 ± 0.0 | 82 | 12 | 6 | 0.01 |
| *H. leopoldo-horstii* | 2.7 ± 1.4 | 60 | 14 | 26 | 0.7 ± 0.2 | 83 | 14 | 3 | 0.26 |
| *H. rosea* | 0.5 ± 0.4 | 55 | 24 | 21 | 0.6 ± 0.2 | 70 | 15 | 15 | 0.19 |
| *H. stellata* | 0.6 ± 0.3 | 39 | 43 | 18 | 1.5 ± 1.3 | 87 | 5 | 8 | 0.08 |
| *H. utriculosa* | 0.5 ± 0.2 | 55 | 34 | 11 | 1.4 ± 0.2 | 83 | 9 | 8 | 0.02 |
| *Lemeltonia narthecioides* | 0.5 ± .04 | 79 | 13 | 8 | 1.7 ± 0.8 | 98 | 0 | 2 | 0.02 |
| *L. scaligera* | 0.4 ± 0.2 | 23 | 66 | 11 | 0.3 ± 0.2 | 91 | 1 | 8 | 0.00 |
| *Neoregelia ampullacea* | 6.1 ± 1.5 | 77 | 17 | 6 | 3.0 ± 1.2 | 68 | 21 | 11 | 0.26 |
| *N. carolinae* | 3.7 ± 2.5 | 74 | 10 | 16 | 2.8 ± 1.3 | 80 | 12 | 8 | 0.09 |
| *N. compacta* | 1.5 ± 0.3 | 32 | 51 | 17 | 1.1 ± 0.3 | 43 | 13 | 44 | 0.38 |
| *N. farinosa* | 0.4 ± 0.1 | 2 | 68 | 30 | 3.9 ± 1.6 | 64 | 18 | 18 | 0.04 |
| *N. fosteriana* | 1.0 ± 0.2 | 85 | 9 | 6 | 0.6 ± 0.1 | 78 | 17 | 5 | 0.10 |
| *N. johannis* | 0.3 ± 0.1 | 47 | 31 | 22 | 0.3 ± 0.2 | 85 | 0 | 15 | 0.38 |
| *N. kautskyi* | 1.9 ± 0.7 | 67 | 19 | 14 | 0.3 ± 0.0 | 51 | 27 | 22 | 0.03 |
| *N. laevis* | 0.7 ± 0.2 | 74 | 11 | 15 | 0.6 ± 0.2 | 54 | 34 | 12 | 0.55 |
| *N. martinellii* | 0.8 ± 0.4 | 68 | 16 | 16 | 0.8 ± 0.1 | 68 | 19 | 13 | 0.71 |
| *N. olens* | 0.7 ± 0.3 | 45 | 29 | 26 | 0.7 ± 0.1 | 54 | 41 | 5 | 0.23 |
| *N. pineliana* | 0.9 ± 0.0 | 63 | 31 | 6 | 0.6 ± 0.2 | 75 | 0 | 25 | 0.27 |
| *N. seideliana* | 4.1 ± 0.5 | 28 | 48 | 24 | 0.4 ± 0.1 | 51 | 22 | 27 | 0.14 |
| *N. wilsoniana* | 0.3 ± 0.2 | 21 | 50 | 29 | 0.4 ± 0.1 | 76 | 14 | 10 | 0.07 |
| *Nidularium amazonicum* | 2.3 ± 0.4 | 75 | 15 | 10 | 2.7 ± 0.8 | 61 | 18 | 21 | 0.43 |
| *Nid. innocentii* | 4.1 ± 1.3 | 89 | 7 | 4 | 4.6 ± 0.8 | 57 | 10 | 33 | 0.29 |
| *Nid. procerum* | 4.8 ± 1.1 | 91 | 4 | 5 | 4.3 ± 1.2 | 58 | 31 | 11 | 0.22 |
| *Nid. purpureum* | 2.0 ± 0.2 | 90 | 4 | 6 | 1.1 ± 0.1 | 64 | 18 | 16 | 0.85 |
| *Nid. rutilans* | 0.5 ± 0.4 | 57 | 22 | 21 | 1.1 ± 0.0 | 61 | 30 | 9 | 0.18 |
| *Nid. scheremetiewii* | 2.4 ± 1.0 | 72 | 4 | 24 | 5.0 ± 0.3 | 78 | 19 | 3 | 1.80 |
| *Nid. utriculosum* | 0.6 ± 0.2 | 75 | 25 | 0 | 0.5 ± 0.1 | 86 | 10 | 4 | 0.00 |
| *Pitcairnia bromeliifolia* | 0.5 ± 0.3 | 58 | 42 | 0 | 2.0 ± 0.1 | 86 | 0 | 14 | 0.02 |
| *Pit. chiapensis* | 0.4 ± 0.0 | 80 | 14 | 6 | 1.7 ± 0.3 | 86 | 7 | 7 | 0.10 |
| *Pit. chiriquensis* | 0.9 ± 0.2 | 59 | 22 | 19 | 1.6 ± 0.2 | 91 | 0 | 9 | 0.03 |
| *Pit. grafii* | 0.2 ± 0.2 | 43 | 39 | 18 | 1.4 ± 0.3 | 97 | 3 | 0 | 0.96 |
| ***Pit. recurvata*** | **10.3 ± 1.9** | **95** | **3** | **2** | **7.3 ± 1.4** | **100** | **0** | **0** | **0.94** |
| *Pit. rubronigriflora* | 4.7 ± 1.2 | 92 | 5 | 3 | 3.1 ± 1.1 | 91 | 5 | 4 | 0.15 |
| *Pit. sprucei* | 0.3 ± 0.2 | 54 | 31 | 15 | 1.4 ± 0.7 | 97 | 1 | 2 | 0.08 |
| *Pit. suaveolens* | 1.1 ± 0.2 | 83 | 10 | 7 | 1.1 ± 0.4 | 94 | 2 | 4 | 0.10 |
| *Pit. utcubambensis* | 0.6 ± 0.4 | 74 | 19 | 7 | 1.7 ± 1.5 | 77 | 4 | 19 | 0.34 |
| *Pit. xanthocalyx* | 1.3 ± 0.1 | 80 | 16 | 4 | 1.7 ± 0.1 | 96 | 0 | 4 | 0.00 |
| ***Pseudalcantarea grandis*** | **6.2 ± 1.2** | **98** | **2** | **0** | **5.8 ± 1.1** | **99** | **1** | **0** | **1.16** |
| ***Pse. macropetala*** | **6.7 ± 2.9** | **91** | **7** | **2** | **2.8 ± 0.8** | **74** | **18** | **8** | **0.73** |
| ***Pse. viridiflora*** | **2.8 ± 1.3** | **93** | **5** | **2** | **4.6 ± 1.4** | **98** | **1** | **1** | **2.23** |
| *Puya coerulea var. violacea* | 1.3 ± 0.5 | 91 | 6 | 3 | 0.1 ± 0.0 | 79 | 0 | 21 | 0.00 |
| *P. densiflora* | 0.1 ± 0.0 | 24 | 51 | 25 | 0.3 ± 0.1 | 100 | 0 | 0 | 0.00 |
| ***P. ferruginea*** | **7.8 ± 2.6** | **97** | **2** | **1** | **3.5 ± 0.2** | **100** | **0** | **0** | **0.11** |
| *P. spathacea* | 0.1 ± 0.0 | 29 | 54 | 17 | 0.3 ± 0.0 | 97 | 0 | 3 | 0.00 |
| *Quesnelia edmundoi* | 0.5 ± 0.1 | 46 | 51 | 3 | 2.8 ± 0.7 | 84 | 13 | 4 | 0.35 |
| *Q. lateralis* | 0.2 ± 0.2 | 54 | 26 | 20 | 0.1 ± 0.0 | 66 | 5 | 29 | 0.00 |
| *Q. quesneliana* | 0.2 ± 0.1 | 51 | 32 | 17 | 0.9 ± 0.2 | 67 | 25 | 8 | 0.32 |
| *Tillandsia achyrostachys* | 0.2 ± 0.0 | 13 | 66 | 21 | 0.5 ± 0.1 | 89 | 4 | 7 | 0.04 |
| *T. aeranthos* | 0.2 ± 0.1 | 0 | 94 | 6 | 9.2 ± 2.7 | 81 | 3 | 16 | 0.00 |
| *T. caput-medusae* | 0.1 ± 0.1 | 46 | 46 | 8 | 0.6 ± 0.3 | 83 | 8 | 9 | 0.09 |
| *T. circinnatoides* | 0.6 ± 0.4 | 59 | 10 | 31 | 2.0 ± 1.1 | 83 | 5 | 12 | 0.29 |
| *T. clavigera* | 0.5 ± 0.1 | 17 | 55 | 28 | 0.3 ± 0.1 | 87 | 0 | 13 | 0.00 |
| *T. concolor* | 0.5 ± 0.2 | 31 | 43 | 26 | 1.5 ± 0.4 | 83 | 4 | 13 | 0.28 |
| *T. flabellata* | 0.3 ± 0.2 | 30 | 44 | 26 | 1.7 ± 0.6 | 96 | 1 | 3 | 0.10 |
| *T. foliosa* | 0.6 ± 0.2 | 46 | 44 | 10 | 0.3 ± 0.1 | 80 | 8 | 12 | 0.03 |
| *T. funckiana* | 1.5 ± 0.7 | 79 | 15 | 6 | 0.3 ± 0.1 | 66 | 18 | 16 | 0.19 |
| *T. gerdae* | 0.4 ± 0.3 | 35 | 43 | 22 | 3.9 ± 1.7 | 90 | 3 | 7 | 0.28 |
| ***T. heterophylla*** | **2.6 ± 0.1** | **96** | **3** | **1** | **1.6 ± 0.3** | **95** | **2** | **3** | **0.50** |
| *T. ionantha* | 0.2 ± 0.1 | 65 | 24 | 11 | 0.5 ± 0.1 | 80 | 7 | 13 | 0.22 |
| *T. ixioides* | 0.4 ± 0.1 | 2 | 77 | 21 | 0.7 ± 0.2 | 87 | 0 | 13 | 0.06 |
| *T. makoyana* | 1.4 ± 0.3 | 75 | 10 | 15 | 0.7 ± 0.2 | 70 | 24 | 6 | 0.41 |
| *T. malzinei* | 0.3 ± 0.2 | 51 | 38 | 11 | 0.5 ± 0.1 | 84 | 4 | 12 | 0.10 |
| *T. polystachia* | 0.4 ± 0.2 | 13 | 65 | 22 | 0.6 ± 0.3 | 63 | 4 | 33 | 0.04 |
| *T. ponderosa* | 0.6 ± 0.1 | 88 | 9 | 3 | 0.2 ± 0.1 | 87 | 0 | 13 | 0.09 |
| *T. propagulifera* | 1.1 ± 0.6 | 39 | 32 | 29 | 0.2 ± 0.0 | 100 | 0 | 0 | 0.14 |
| ***T. rauhii*** | **6.5 ± 2.4** | **75** | **23** | **2** | **2.8 ± 0.1** | **98** | **0** | **2** | **0.00** |
| *T. roland-gosselinii* | 0.5 ± 0.1 | 43 | 41 | 16 | 0.4 ± 0.1 | 97 | 0 | 3 | 0.02 |
| *T. tricolor* | 2.1 ± 0.6 | 50 | 42 | 8 | 0.9 ± 0.2 | 92 | 4 | 4 | 0.08 |
| ***Vriesea bituminosa*** | **9.6 ± 3.3** | **74** | **25** | **1** | **2.4 ± 0.4** | **100** | **0** | **0** | **0.65** |
| *V. bleheri* | 0.1 ± 0.0 | 23 | 54 | 23 | 0.5 ± 0.2 | 78 | 1 | 21 | 0.00 |
| *V. drepanocarpa* | 0.5 ± 0.5 | 37 | 43 | 20 | 1.4 ± 0.2 | 93 | 0 | 7 | 0.00 |
| *V. dubia* | 1.0 ± 0.3 | 89 | 9 | 2 | 0.2 ± 0.1 | 65 | 3 | 32 | 0.09 |
| *V. eltoniana* | 1.3 ± 0.8 | 52 | 42 | 6 | 0.6 ± 0.1 | 66 | 6 | 28 | 0.08 |
| ***V. fenestralis*** | **7.4 ± 1.8** | **95** | **3** | **2** | **6.9 ± 0.7** | **86** | **9** | **5** | **1.39** |
| *V. friburgensis tucumanensis* | 2.1 ± 0.1 | 94 | 6 | 0 | 1.4 ± 0.1 | 55 | 14 | 31 | 0.13 |
| *V. guttata* | 0.3 ± 0.2 | 11 | 48 | 41 | 1.2 ± 0.3 | 90 | 4 | 6 | 0.44 |
| *V. maxoniana* | 0.1 ± 0.1 | 18 | 54 | 28 | 0.5 ± 0.1 | 80 | 4 | 16 | 0.00 |
| ***V. nanuzae*** | **4.6 ± 1.1** | **97** | **2** | **1** | **1.9 ± 0.3** | **96** | **0** | **4** | **0.09** |
| ***V. racinae*** | **3.0 ± 0.1** | **13** | **30** | **57** | **3.1 ± 0.6** | **90** | **1** | **9** | **0.00** |
| *V. saundersii* | 0.3 ± 0.1 | 5 | 64 | 31 | 0.2 ± 0.1 | 93 | 4 | 3 | 0.00 |
| *V. scalaris* | 1.0 ± 0.0 | 50 | 22 | 28 | 1.7 ± 0.4 | 90 | 1 | 9 | 0.04 |
| ***V. unilateralis*** | **7.4 ± 1.4** | **93** | **5** | **2** | **1.7 ± 0.1** | **96** | **4** | **0** | **0.88** |
| *Wallisia cyanea* | 0.4 ± 0.2 | 26 | 35 | 39 | 0.6 ± 0.3 | 87 | 11 | 2 | 0.00 |
| *W. lindeniana* | 0.2 ± 0.0 | 17 | 43 | 40 | 0.7 ± 0.4 | 76 | 14 | 10 | 0.03 |
| *W. pretiosa* | 0.7 ± 0.2 | 68 | 32 | 0 | 1.2 ± 0.2 | 87 | 0 | 13 | 0.07 |
| ***Werauhia gladioliflora*** | **8.7 ± 1.5** | **94** | **5** | **1** | **4.6 ± 0.7** | **95** | **4** | **1** | **0.95** |
| ***Wer. nutans*** | **8.1 ± 2.0** | **94** | **5** | **1** | **8.2 ± 0.8** | **89** | **7** | **4** | **0.00** |
| *Wer. patzeltii* | 0.6 ± 0.1 | 46 | 43 | 11 | 0.9 ± 0.3 | 86 | 14 | 0 | 0.15 |
| ***Wer. pectinata*** | **14.0 ± 3.5** | **97** | **2** | **1** | **8.5 ± 1.3** | **82** | **16** | **2** | **10.78** |
| ***Wer. sanguinolenta*** | **13.2 ± 0.6** | **95** | **3** | **2** | **4.2 ± 1.0** | **88** | **2** | **10** | **0.30** |
| ***Wer. werckleana*** | **22.5 ± 1.8** | **97** | **2** | **1** | **3.4 ± 1.4** | **100** | **0** | **0** | **0.25** |

Bold type = bat-pollinated bromeliads. n.d. = not detectable.
