# Supplementary material for: What Do Nectarivorous Bats Like? Nectar Composition in Bromeliaceae With Special Emphasis on Bat-Pollinated Species
Source: Front Plant Sci. 2019 Feb 21;10:205. doi: 10.3389/fpls.2019.00205 (PMC6393375; doi:10.3389/fpls.2019.00205)
Supplement: Supplementary file 5 [file Table_5.docx]

Supplementary Material

What do nectarivorous bats like? Nectar composition in Bromeliaceae with special emphasis on bat-pollinated species

**Author: Thomas Göttlinger, Michael Schwerdtfeger, Kira Tiedge, Gertrud Lohaus***

***Correspondence:** Gertrud Lohaus (lohaus@uni-wuppertal.de)

**Supplementary Table S5:** Results of the PERMANOVA taking into account the individual components of nectar (corresponding Table 1).R^2^ describes the influence of the pollinator, the genus and the botanical garden on the nectar composition.

|  | **Degrees of freedom (df)** | **Pseudo-F (F)** | **R^2^** | ***p*-value** |
| --- | --- | --- | --- | --- |
| *Sugar [mM]* | | | | |
| Pollinator | 1 | 85.90 | 0.41 | 0.001 *** |
| Genus | 6 | 2.13 | 0.06 | 0.019 * |
| Bot. garden | 3 | 1.68 | 0.02 | 0.125 |
| Pollinator x Genus | 6 | 2.52 | 0.07 | 0.007 ** |
| Pollinator x Bot. garden | 3 | 1.92 | 0.03 | 0.079 |
| Genus x Bot. garden | 1 | 2.85 | 0.01 | 0.064 |
| Pollinator x Genus x Bot. garden | 1 | 14.38 | 0.07 | 0.001 *** |
| Residuals | 68 |  | 0.32 |  |
| Total | 89 |  | 1.00 |  |
| *Amino acids [mM]* | | | | |
| Pollinator | 1 | 26.35 | 0.03 | 0.001 *** |
| Genus | 6 | 54.20 | 0.37 | 0.001 *** |
| Bot. garden | 3 | 8.62 | 0.03 | 0.001 *** |
| Pollinator x Genus | 6 | 61.05 | 0.42 | 0.001 *** |
| Pollinator x Bot. garden | 3 | 6.65 | 0.02 | 0.001 *** |
| Genus x Bot. garden | 1 | 21.11 | 0.02 | 0.001 *** |
| Pollinator x Genus x Bot. garden | 1 | 19.24 | 0.02 | 0.001 *** |
| Residuals | 68 |  | 0.08 |  |
| Total | 89 |  | 1.00 |  |
| *Inorganic ions and organic acids [mM]* | | | | |
| Pollinator | 1 | 73.03 | 0.34 | 0.001 *** |
| Genus | 6 | 6.28 | 0.18 | 0.001 *** |
| Bot. garden | 3 | 5.06 | 0.07 | 0.002 ** |
| Pollinator x Genus | 6 | 2.21 | 0.06 | 0.011 * |
| Pollinator x Bot. garden | 3 | 1.49 | 0.02 | 0.198 |
| Genus x Bot. garden | 1 | 1.97 | 0.01 | 0.144 |
| Pollinator x Genus x Bot. garden | 1 | 1.20 | 0.01 | 0.266 |
| Residuals | 68 |  | 0.32 |  |
| Total | 89 |  | 1.00 |  |
