# Supplementary material for: What Do Nectarivorous Bats Like? Nectar Composition in Bromeliaceae With Special Emphasis on Bat-Pollinated Species
Source: Front Plant Sci. 2019 Feb 21;10:205. doi: 10.3389/fpls.2019.00205 (PMC6393375; doi:10.3389/fpls.2019.00205)
Supplement: Supplementary file 6 [file Table_6.docx]

Supplementary Material

What do nectarivorous bats like? Nectar composition in Bromeliaceae with special emphasis on bat-pollinated species

**Author: Thomas Göttlinger, Michael Schwerdtfeger, Kira Tiedge, Gertrud Lohaus***

***Correspondence:** Gertrud Lohaus (lohaus@uni-wuppertal.de)

**Supplementary Table S6:** Sugar-ratios in nectar of bromeliad species collected during fieldwork in Bolivia and Mexico or in botanical gardens in Germany. Data from field plants were derived from (Krömer et al., 2008) and data from greenhouse plants were derived from Supplementary Table S2 (this work).

|  | **Field plants** | | **Greenhouse plants** | |
| --- | --- | --- | --- | --- |
| **Species** | ***n*** | **Sucrose-to-hexoses ratio** | ***n*** | **Sucrose-to-hexoses ratio** |
| **chiropterophilus** | | | | |
| *Guzmania killipiana* | 1 | 0.6 | 3 | 0.4 ± 0.2 |
| *Tillandsia heterophylla* | 2 | 0.3 ± 0.0 | 3 | 0.3 ± 0.0 |
| *Tillandsia viridiflora (Pseudalcantarea viridiflora)* | 7 | 0.3 ± 0.1 | 3 | 0.5 ± 0.1 |
| *Werauhia nutans* | 5 | 0.6 ± 0.1 | 3 | 0.5 ± 0.2 |
| **trochilophilus** | | | | |
| *Guzmania melinonis* | 1 | 2.6 | 3 | 1.5 ± 0.2 |

**References**

Krömer, T., Kessler, M., Lohaus, G., and Schmidt-Lebuhn, A.N. (2008). Nectar sugar composition and concentration in relation to pollination syndromes in Bromeliaceae. Plant Biol (Stuttg) 10, 502–511. doi: 10.1111/j.1438-8677.2008.00058.x
