# Supplementary material for: What Do Nectarivorous Bats Like? Nectar Composition in Bromeliaceae With Special Emphasis on Bat-Pollinated Species
Source: Front Plant Sci. 2019 Feb 21;10:205. doi: 10.3389/fpls.2019.00205 (PMC6393375; doi:10.3389/fpls.2019.00205)
Supplement: Supplementary file 7 [file Data_Sheet_1.pdf]

*Supplementary Material*

# What do nectarivorous bats like? Nectar composition in Bromeliaceae with special emphasis on bat-pollinated species

**Author: Thomas Göttlinger, Michael Schwerdtfeger, Kira Tiedge, Gertrud Lohaus***

***Correspondence:** Gertrud Lohaus (lohaus@uni-wuppertal.de)

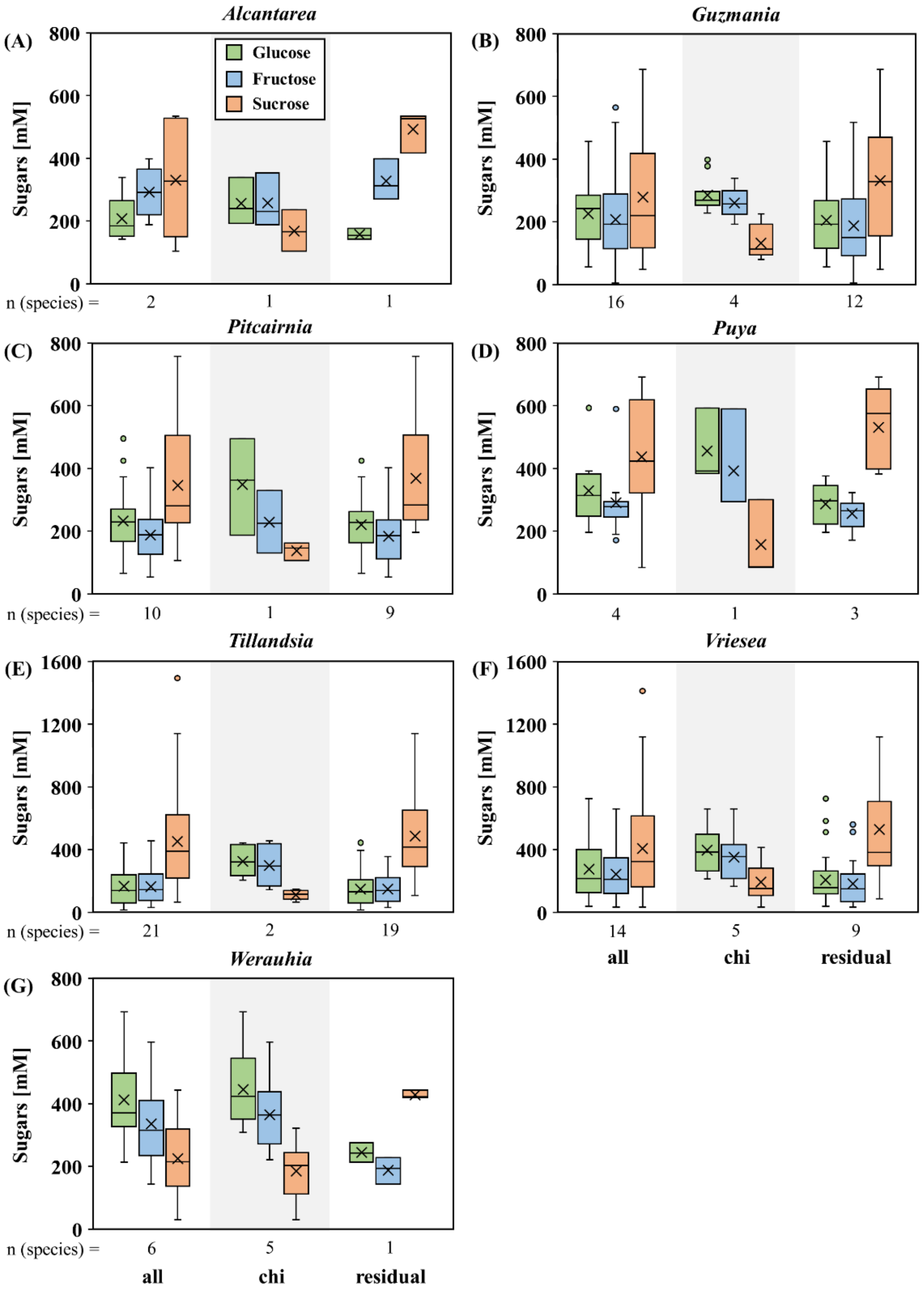


**Supplementary Figure S1:** Concentration of sugars (glucose, fructose, sucrose) in nectar of seven genera of Bromeliaceae (Alcantarea (A), Guzmania (B), Pitcairnia (C), Puya (D), Tillandsia (E), Vriesea (F), Werauhia (G)) which include bat-pollinated species. The box plots show medians (horizontal line in box) and means (x in box).
