# Supplementary material for: What Do Nectarivorous Bats Like? Nectar Composition in Bromeliaceae With Special Emphasis on Bat-Pollinated Species
Source: Front Plant Sci. 2019 Feb 21;10:205. doi: 10.3389/fpls.2019.00205 (PMC6393375; doi:10.3389/fpls.2019.00205)
Supplement: Supplementary file 8 [file Data_Sheet_2.pdf]

Supplementary Material

What do nectarivorous bats like? Nectar composition in Bromeliaceae with special emphasis on bat-pollinated species

Author: Thomas Göttlinger, Michael Schwerdtfeger, Kira Tiedge, Gertrud Lohaus*

***Correspondence:** Gertrud Lohaus (lohaus@uni-wuppertal.de)

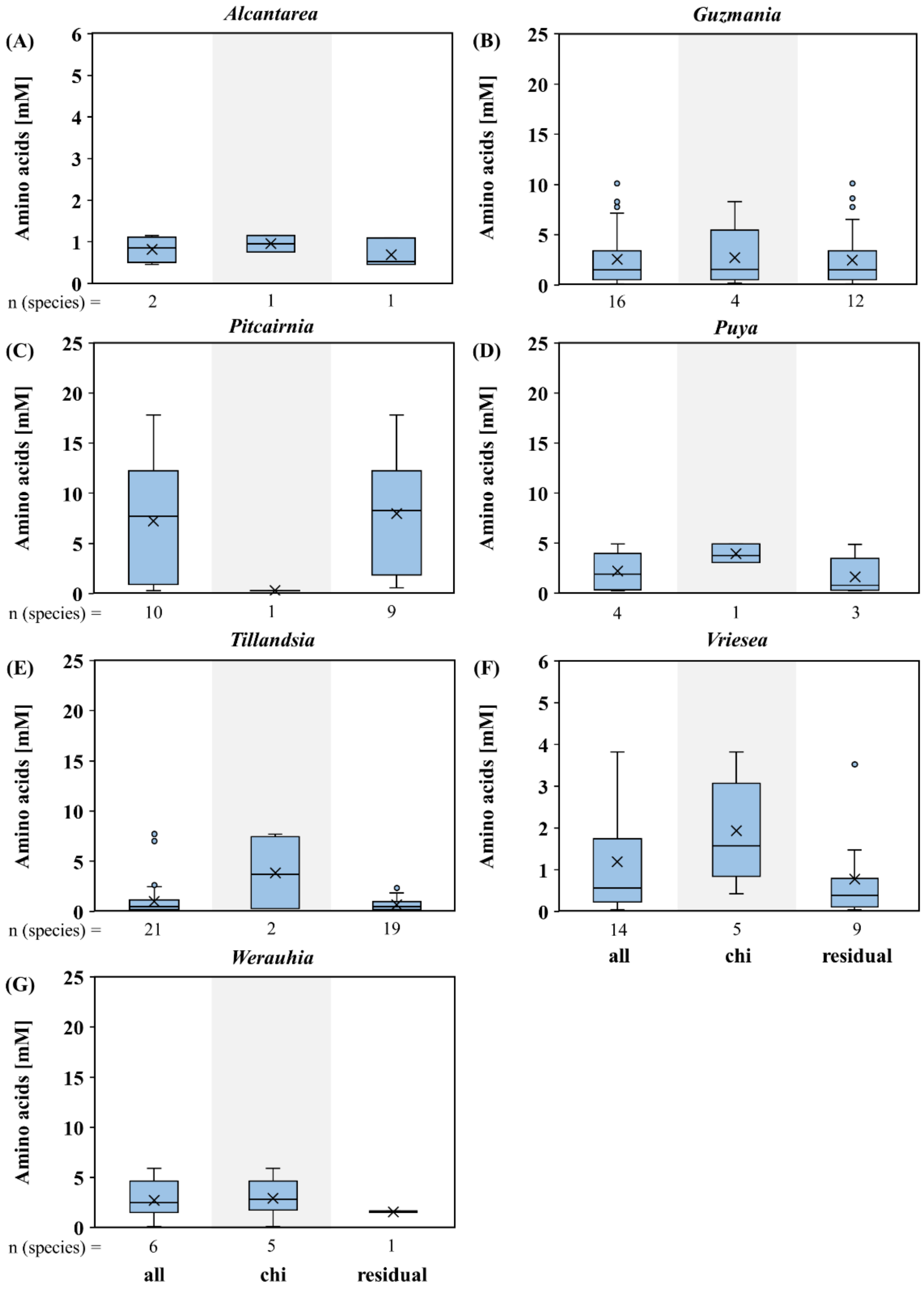

Supplementary Figure S2: Concentration of amino acids (ala, arg, asn, asp, gaba, gln, glu, gly, his, iso, leu, lys, met, phe, pro, ser, thr, trp, tyr, val) in nectar of seven genera of Bromeliaceae (Alcantarea (A), Guzmania (B)**,** Pitcairnia (C), Puya (D), Tillandsia (E), Vriesea (F), Werauhia (G)), which include bat-pollinated species. The box plots show medians (horizontal line in box) and means (x in box).
