# Supplementary material for: What Do Nectarivorous Bats Like? Nectar Composition in Bromeliaceae With Special Emphasis on Bat-Pollinated Species
Source: Front Plant Sci. 2019 Feb 21;10:205. doi: 10.3389/fpls.2019.00205 (PMC6393375; doi:10.3389/fpls.2019.00205)
Supplement: Supplementary file 11 [file Data_Sheet_5.pdf]

Supplementary Material

What do nectarivorous bats like? Nectar composition in Bromeliaceae with special emphasis on bat-pollinated species

Author: Thomas Göttlinger, Michael Schwerdtfeger, Kira Tiedge, Gertrud Lohaus*

***Correspondence:** Gertrud Lohaus (lohaus@uni-wuppertal.de)

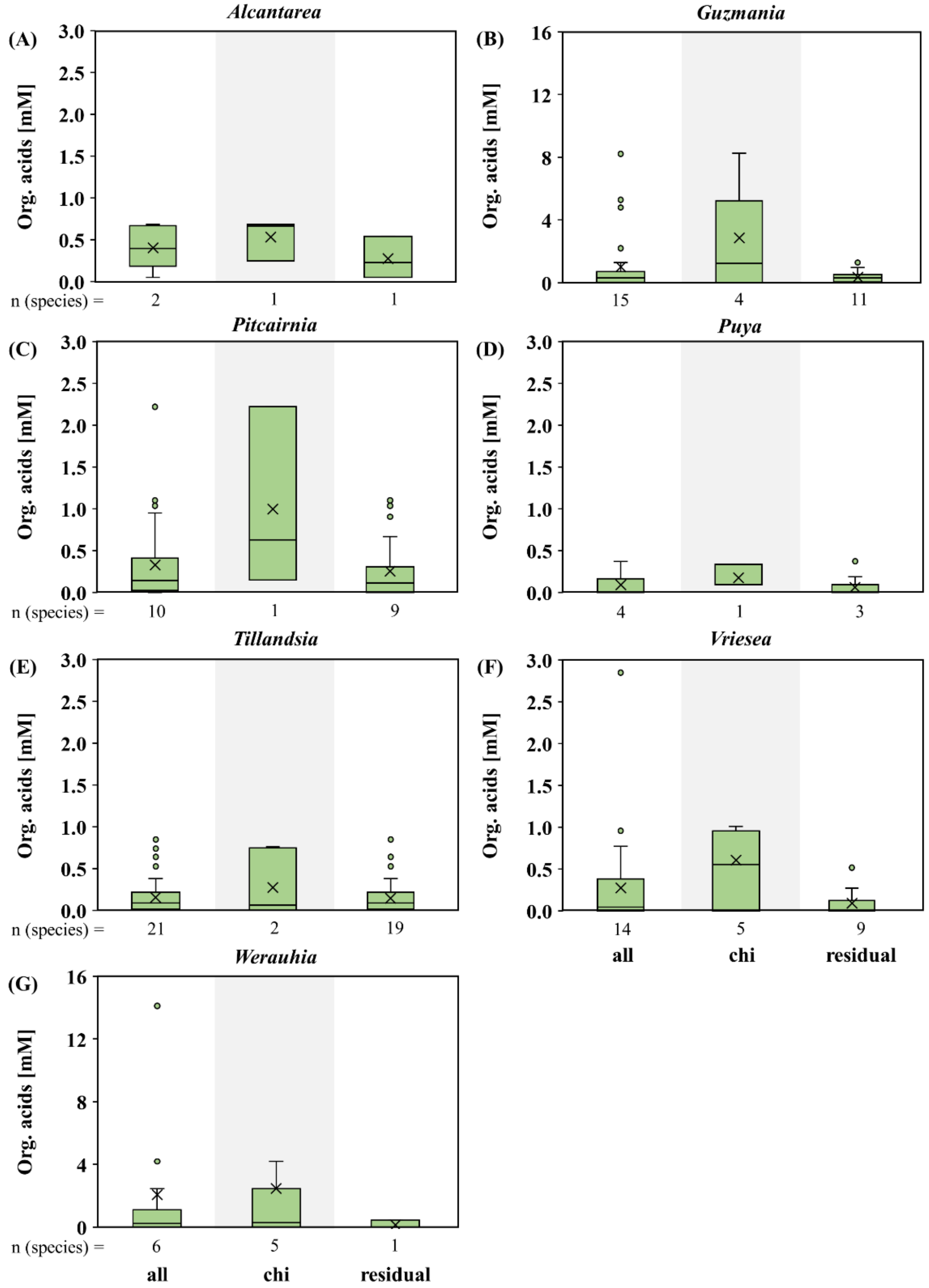

Supplementary Figure S5: Concentration of organic acids (malate, citrate) in nectar of seven Bromeliaceae genera (Alcantarea (A), Guzmania (B), Pitcairnia (C), Puya (D), Tillandsia (E), Vriesea (F), Werauhia (G)), which include bat-pollinated species. The box plots show medians (horizontal line in box) and means (x in box).
