# Supplementary material for: What Do Nectarivorous Bats Like? Nectar Composition in Bromeliaceae With Special Emphasis on Bat-Pollinated Species
Source: Front Plant Sci. 2019 Feb 21;10:205. doi: 10.3389/fpls.2019.00205 (PMC6393375; doi:10.3389/fpls.2019.00205)
Supplement: Supplementary file 12 [file Data_Sheet_6.pdf]

Supplementary Material

What do nectarivorous bats like? Nectar composition in Bromeliaceae with special emphasis on bat-pollinated species

Author: Thomas Göttlinger, Michael Schwerdtfeger, Kira Tiedge, Gertrud Lohaus*

***Correspondence:** Gertrud Lohaus (lohaus@uni-wuppertal.de)

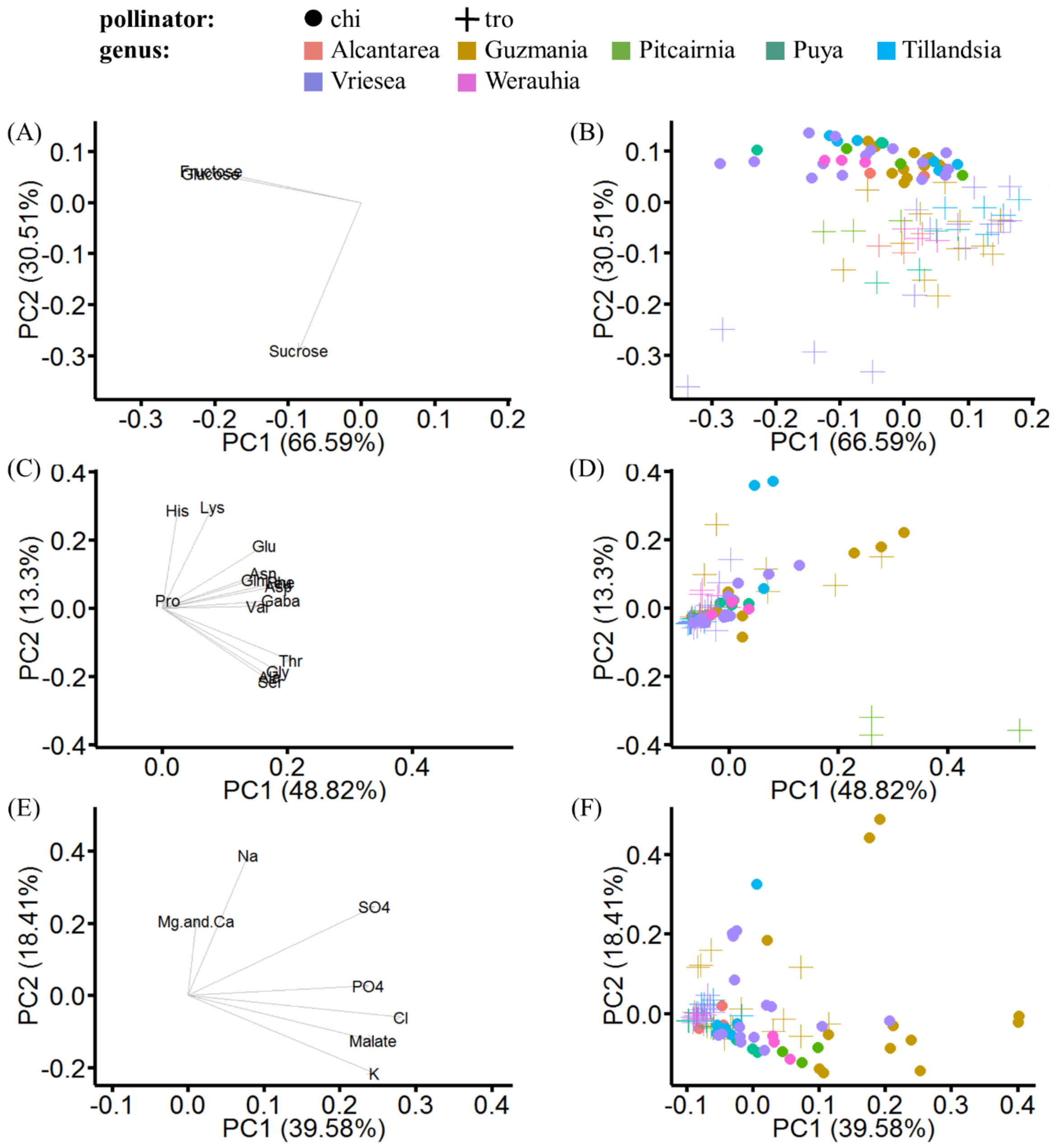

Supplementary Figure S6: Loadings and scatterplot of PCA in rotated space (pollinator and taxonomic groups, growth site). In the PCA, the statistically analyzed samples were selected according to their pollinator and to their taxonomic groups (genera). A, C, E: Loading plot, which illustrate the original variables (A: sugars, C: amino acids, E: inorganic ions and organic acids) loaded as vectors in PCA space. B, D, F: Scatterplot of PCA, in which the data (B: sugars, D: amino acids, F: inorganic ions and organic acids) are grouped by pollinator (markings) and genus (colors).
