# Supplementary material for: What Do Nectarivorous Bats Like? Nectar Composition in Bromeliaceae With Special Emphasis on Bat-Pollinated Species
Source: Front Plant Sci. 2019 Feb 21;10:205. doi: 10.3389/fpls.2019.00205 (PMC6393375; doi:10.3389/fpls.2019.00205)
Supplement: Supplementary file 13 [file Data_Sheet_7.pdf]

Supplementary Material

What do nectarivorous bats like? Nectar composition in Bromeliaceae with special emphasis on bat-pollinated species

Author: Thomas Göttlinger, Michael Schwerdtfeger, Kira Tiedge, Gertrud Lohaus*

***Correspondence:** Gertrud Lohaus (lohaus@uni-wuppertal.de)

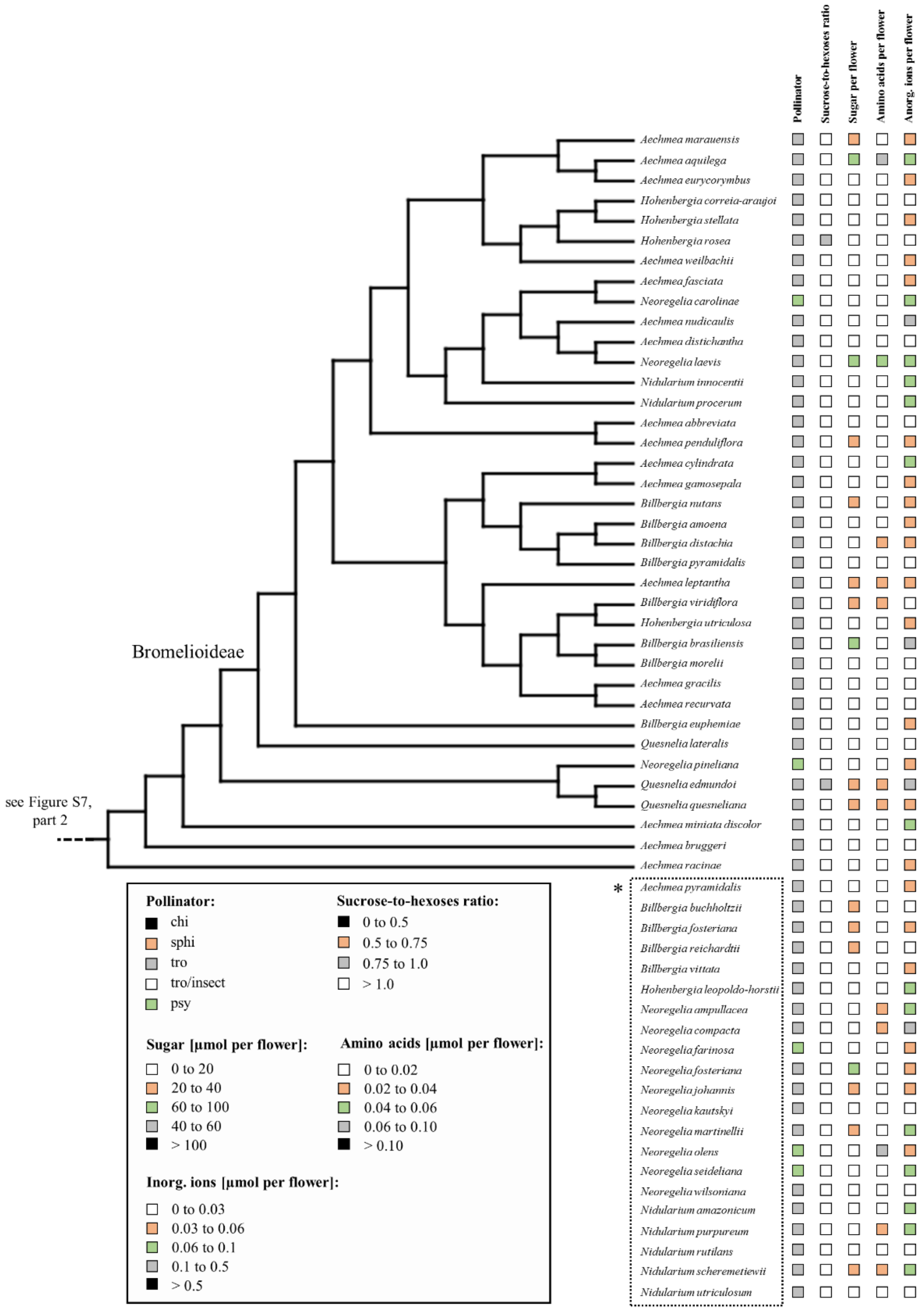

Supplementary Figure S7: Simplified phylogram of all analyzed Bromeliaceae species combining molecular and morphological findings. The schematic phylogenetic tree is based on 23 different phylogenetic investigations

(Faria et al., 2004; Barfuss et al., 2005; Givnish, 2007; Horres et al., 2007; Hornung-Leoni et al., 2008; Almeida et al., 2009; Rex et al., 2009; Schulte et al., 2009; Chew et al., 2010; Jabaily and Sytsma, 2010; Sass and Specht, 2010; Givnish et al., 2011; Gomes-da-Silva et al., 2012; Versieux et al., 2012; Escobedo-Sarti et al., 2013; Givnish et al., 2014; Costa et al., 2015; Evans et al., 2015; Barfuss et al., 2016; Pinzón et al., 2016; Schütz et al., 2016; Gomes-da-Silva and Souza-Chies, 2018; Moura et al., 2018). The phylogenetic tree was created using Mesquite 3.51. * No molecular phylogenetic data available. Bold type = bat-pollinated bromeliads.

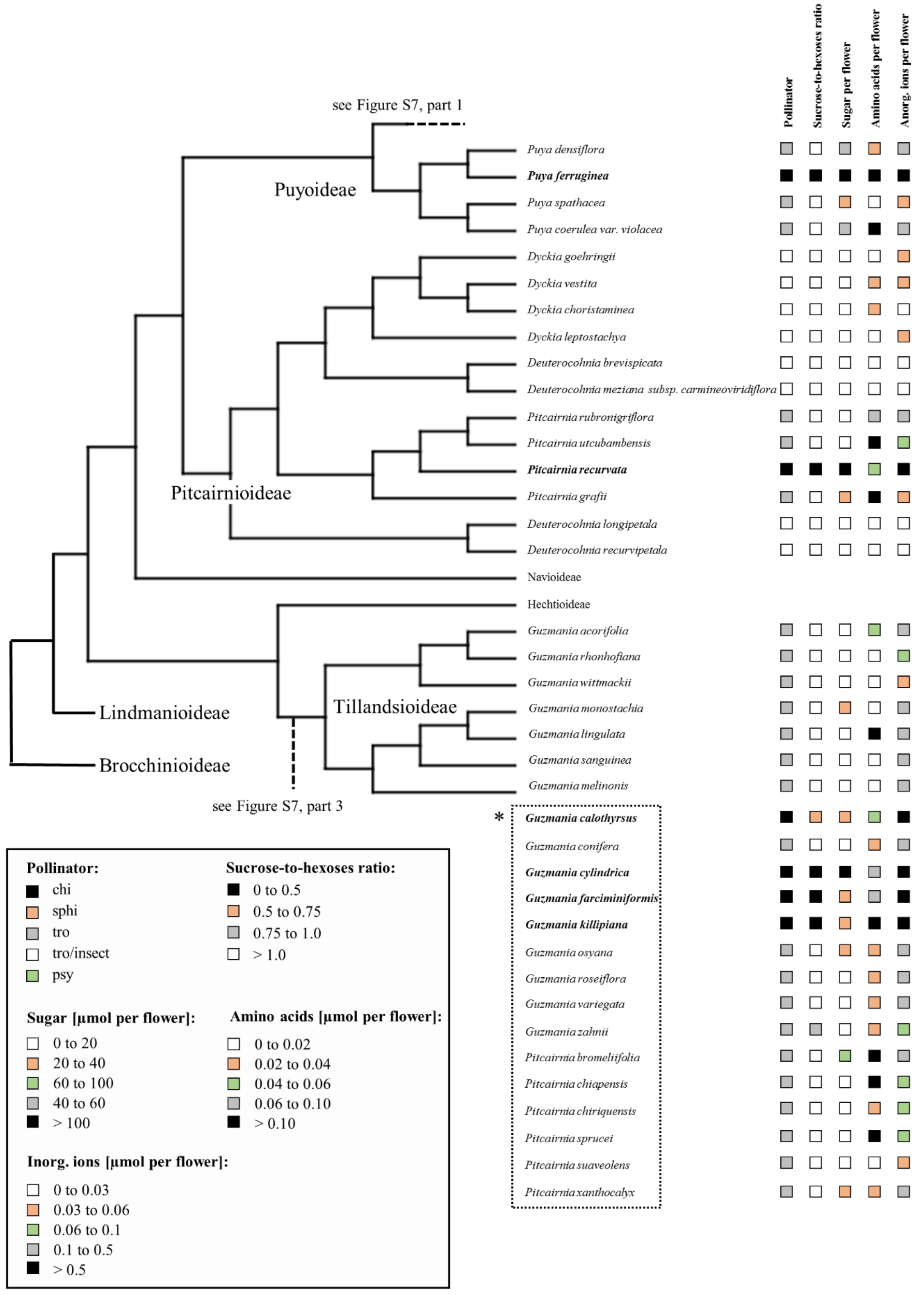

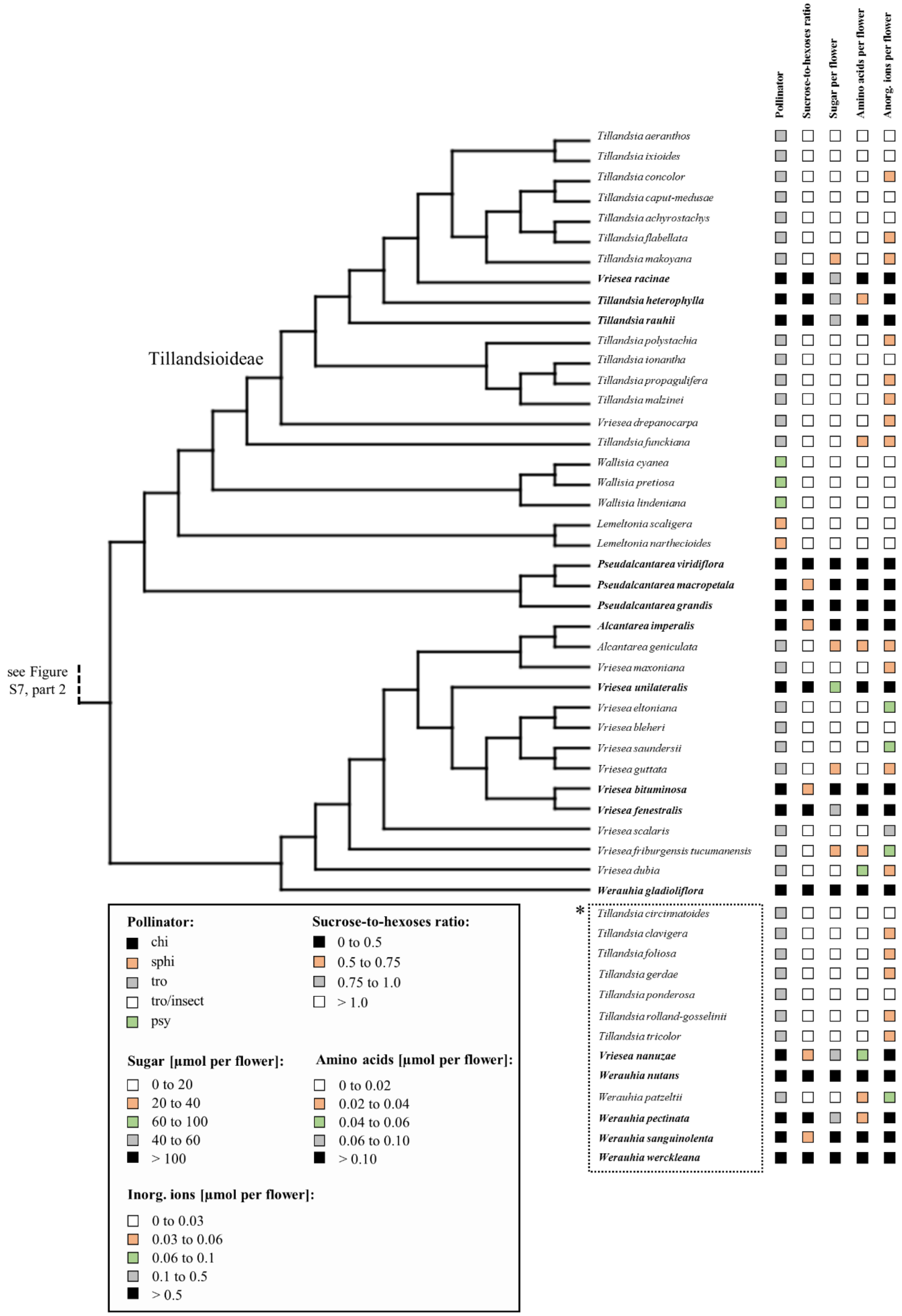
Pollinator
Sucrose-to-hexoses ratio
Sugar per flower
Amino acids per flower
Anorg. ions per flower
Tillandsioideae
see Figure S7, part 2
Tillandsia aeranthos
Tillandsia ixioides
Tillandsia concolor
Tillandsia caput-medusae
Tillandsia achyrostachys
Tillandsia flabellata
Tillandsia makoyana
Vriesea racinae
Tillandsia heterophylla
Tillandsia rauhii
Tillandsia polystachia
Tillandsia ionantha
Tillandsia propagulifera
Tillandsia malzinei
Vriesea drepanocarpa
Tillandsia funckiana
Wallisia cyanea
Wallisia pretiosa
Wallisia lindeniana
Lemeltonia scaligera
Lemeltonia narthecioides
Pseudalcantarea viridiflora
Pseudalcantarea macropetala
Pseudalcantarea grandis
Alcantarea imperalis
Alcantarea geniculata
Vriesea maxoniana
Vriesea unilateralis
Vriesea eltoniana
Vriesea bleheri
Vriesea saundersii
Vriesea guttata
Vriesea bituminosa
Vriesea fenestralis
Vriesea scalaris
Vriesea friburgensis tucumanensis
Vriesea dubia
Werauhia gladioliflora
*
Tillandsia circinnatoides
Tillandsia clavigera
Tillandsia foliosa
Tillandsia gerdae
Tillandsia ponderosa
Tillandsia rolland-gosselinii
Tillandsia tricolor
Vriesea nanuzae
Werauhia nutans
Werauhia patzeltii
Werauhia pectinata
Werauhia sanguinolenta
Werauhia werckleana
Pollinator:
chi
sphi
tro
tro/insect
psy
Sucrose-to-hexoses ratio:
0 to 0.5
0.5 to 0.75
0.75 to 1.0
> 1.0
Sugar [µmol per flower]:
0 to 20
20 to 40
60 to 100
40 to 60
> 100
Amino acids [µmol per flower]:
0 to 0.02
0.02 to 0.04
0.04 to 0.06
0.06 to 0.10
> 0.10
Inorg. ions [µmol per flower]:
0 to 0.03
0.03 to 0.06
0.06 to 0.1
0.1 to 0.5
> 0.5

References

Almeida, V.R., Ferreira da Costa, A., Mantovani, A., Gonçalves-Esteves, V., Oliveira, R., and Forzza, R.C. (2009). Morphological phylogenetics of *Quesnelia* (Bromeliaceae, Bromelioideae). Systematic Botany 34, 660–672. doi: 10.1600/036364409790139619

Barfuss, M.H.J., Samuel, R., Till, W., and Stuessy, T.F. (2005). Phylogenetic relationships in subfamily Tillandsioideae (Bromeliaceae) based on DNA sequence data from seven plastid regions. Am J Bot 92, 337–351. doi: 10.3732/ajb.92.2.337

Barfuss, M.H.J., Till, W., Leme, E., Pinzón, J. P., Manzanare, J. M., Halbritter, H., et al. (2016). Taxonomic revision of Bromeliaceae subfam. Tillandsioideae based on a multi-locus DNA sequence phylogeny and morphology. Phytotaxa 279, 1. doi: 10.11646/phytotaxa.279.1.1

Chew, T., Luna, E. de, and González, D. (2010). Phylogenetic relationships of the Pseudobulbous *Tillandsia* species (Bromeliaceae) inferred from cladistic analyses of ITS 2, 5.8S ribosomal RNA gene, and ETS sequences. Systematic Botany 35, 86–95. doi: 10.1600/036364410790862632

Costa, A.F., Gomes-da-Silva, J., and Wanderley, M.D. (2015). Vriesea (Bromeliaceae, Tillandsioideae): A cladistic analysis of eastern Brazilian species based on morphological characters. Rodriguésia 66, 429–440. doi: 10.1590/2175-7860201566211

Escobedo-Sarti, J., Ramírez, I., Leopardi, C., Carnevali, G., Magallón, S., Duno, R., et al. (2013). A phylogeny of Bromeliaceae (Poales, Monocotyledoneae) derived from an evaluation of nine supertree methods. Journal of Systematics Evolution 51, 743–757. doi: 10.1111/jse.12044

Evans, T.M., Jabaily, R.S., Faria, A.P., Sousa, L.O., Wendt, T., and Brown, G.K. (2015). Phylogenetic relationships in Bromeliaceae subfamily Bromelioideae based on chloroplast DNA sequence data. Systematic Botany 40, 116–128. doi: 10.1600/036364415X686413

Faria, A.P., Brown, G.K., and Wendt, T. (2004). Cladistic relationships of *Aechmea* (Bromeliaceae, Bromelioideae) and allied genera. Annals of the Missouri Botanical Garden 91, 303–319.

Givnish, T.J. (2007). Phylogeny, adaptive radiation and historical biogeography of Bromeliaceae inferred from ndhF sequence data. Aliso: A Journal of Systematic and Evolutionary Botany: Vol. 23: Iss. 1, Article 4.

Givnish, T.J., Barfuss, M.H.J., Ee, B. V., Riina, R., Schulte, K., Horres, R., et al. (2011). Phylogeny, adaptive radiation, and historical biogeography in Bromeliaceae: Insights from an eight-locus plastid phylogeny. Am J Bot 98, 872–895. doi: 10.3732/ajb.1000059

Givnish, T.J., Barfuss, M.H.J., van Ee, B., Riina, R., Schulte, K., Horres, R., et al. (2014). Adaptive radiation, correlated and contingent evolution, and net species diversification in Bromeliaceae. Mol Phylogenet Evol 71, 55–78. doi: 10.1016/j.ympev.2013.10.010

Gomes-da-Silva, J., Alves da Costa Vargens, F., do Carmo de Oliveira Arruda, R., and Ferreira da Costa, A. (2012). A morphological cladistic analysis of the *Vriesea corcovadensis* group (Bromeliaceae: Tillandsiodeae), with anatomical descriptions: New evidence of the non-monophyly of the genus. Systematic Botany 37, 641–654. doi: 10.1600/036364412X648599

Gomes-da-Silva, J., and Souza-Chies, T.T. (2018). What actually is *Vriesea*? A total evidence approach in a polyphyletic genus of Tillandsioideae (Bromeliaceae, Poales). Cladistics 34, 181–199. doi: 10.1111/cla.12200

Hornung-Leoni, C.T., Sosa, V., and Hornung-Leoni, C.T. (2008). Morphological phylogenetics of *Puya* subgenus *Puya* (Bromeliaceae). Bot J Linn Soc 156, 93–110. doi: 10.1111/j.1095-8339.2007.00740.x

Horres, R., Schulte, K., Weising, K., and Zizka, G. (2007). Systematics of Bromelioideae (Bromeliaceae) - Evidence from molecular and anatomical studies. Aliso 23, 27–43. doi: 10.5642/aliso.20072301.05

Jabaily, R.S., and Sytsma, K.J. (2010). Phylogenetics of *Puya* (Bromeliaceae): Placement, major lineages, and evolution of Chilean species. Am J Bot 97, 337–356. doi: 10.3732/ajb.0900107

Moura, M.N., Forzza, R.C., and Cristiano, M.P. (2018). Reconstruction of ancestral genome size in Pitcairnioideae (Bromeliaceae): What can genome size tell us about the evolutionary history of its five genera? Bot J Linn Soc 186, 321–333. doi: 10.1093/botlinnean/box101

Pinzón, J.P., Ramírez-Morillo, I.M., Carnevali, G., Barfuss, M.H.J., Till, W., Tun, J., et al. (2016). Phylogenetics and evolution of the *Tillandsia utriculata* complex (Bromeliaceae, Tillandsioideae) inferred from three plastid DNA markers and the ETS of the nuclear ribosomal DNA. Bot J Linn Soc 181, 362–390. doi: 10.1111/boj.12425

Rex, M., Schulte, K., Zizka, G., Peters, J., Vásquez, R., Ibisch, P.L., et al. (2009). Phylogenetic analysis of *Fosterella* L.B. Sm. (Pitcairnioideae, Bromeliaceae) based on four chloroplast DNA regions. Mol Phylogenet Evol 51, 472–485. doi: 10.1016/j.ympev.2009.01.001

Sass, C., and Specht, C.D. (2010). Phylogenetic estimation of the core Bromelioids with an emphasis on the genus *Aechmea* (Bromeliaceae). Mol Phylogenet Evol 55, 559–571. doi: 10.1016/j.ympev.2010.01.005

Schulte, K., Barfuss, M.H.J., and Zizka, G. (2009). Phylogeny of Bromelioideae (Bromeliaceae) inferred from nuclear and plastid DNA loci reveals the evolution of the tank habit within the subfamily. Mol Phylogenet Evol 51, 327–339. doi: 10.1016/j.ympev.2009.02.003

Schütz, N., Krapp, F., Wagner, N., and Weising, K. (2016). Phylogenetics of Pitcairnioideae s.s . (Bromeliaceae): Evidence from nuclear and plastid DNA sequence data. Bot J Linn Soc 181, 323–342. doi: 10.1111/boj.12403

Versieux, L.M., Barbará, T., Wanderley, M.D., Calvente, A., Fay, M.F., and Lexer, C. (2012). Molecular phylogenetics of the Brazilian giant bromeliads (Alcantarea, Bromeliaceae): Implications for morphological evolution and biogeography. Mol Phylogenet Evol 64, 177–189. doi: 10.1016/j.ympev.2012.03.015
